# A nonconvex $\hbox{TV}_q-l_1$ regularization model and the ADMM based algorithm

**DOI:** 10.1038/s41598-022-11938-7

**Published:** 2022-05-13

**Authors:** Zhuang Fang, Tang Liming, Wu Liang, Liu Hanxin

**Affiliations:** School of Mathematics and Statistics, Hubei Minzu University, Enshi, 445000 People’s Republic of China

**Keywords:** Applied mathematics, Computational science, Computer science, Information technology, Software

## Abstract

The total variation (TV) regularization with $l_1$ fidelity is a popular method to restore the image contaminated by salt and pepper noise, but it often suffers from limited performance in edge-preserving. To solve this problem, we propose a nonconvex $\hbox{TV}_q-l_1$ regularization model in this paper, which utilizes a nonconvex $l_q$-norm $(0<q<1)$ defined in total variation (TV) domain (called $\hbox{TV}_q$ regularizer) to regularize the restoration, and uses $l_1$ fidelity to measure the noise. Compared to the traditional TV model, the proposed model can more effectively preserve edges and contours since it provides a more sparse representation of the restoration in TV domain. An alternating direction method of multipliers (ADMM) combining with majorization-minimization (MM) scheme and proximity operator is introduced to numerically solve the proposed model. In particular, a sufficient condition for the convergence of the proposed algorithm is provided. Numerical results validate the proposed model and algorithm, which can effectively remove salt and pepper noise while preserving image edges and contours. In addition, compared with several state-of-the-art variational regularization models, the proposed model shows the best performance in terms of peak signal to noise ratio (PSNR) and mean structural similarity index (MSSIM). We can obtain about 0.5 dB PSNR and 0.06 MSSIM improvements against all compared models.

Images are often contaminated by additive noise during the formation, transition or recording process, usually modeled as:

1 $$\begin{aligned} f=u+n, \end{aligned}$$

where *u* is the original true image, *f* is the corresponding noisy version, and *n* represents additive noise. Solving the *u* from the linear system (1) is a classical inverse problem, which is actually an ill-posed problem since the solution of *u* is non-unique and is very sensitive to the initialization. A nature method to address this problem is regularization technique and functional minimization by introducing some prior informations on the restorations^1–5^, usually formulated as:

$$\begin{aligned} \mathop {\min }\limits _u \left\{ {R(u) + \lambda F(f - u)} \right\} ,\;\;{\text{s.t.}}\;\;u \in U, \end{aligned}$$

where *R*(*u*) is regularization term that embodies the priors, $F(f-u)$ is the fidelity term that forces the closeness of the restoration *u* to the observation *f*, *U* is a function space modeling the restoration *u*, and $\lambda >0$ is a tuning parameter that controls the tradeoff between the two terms.

For the **regularization term**, the earliest regularizer is Tikhonov regularization term proposed by Phillips^6^ and Tikhonov^7^ in 1960s, which is defined as a quadric functional of the $l_2$ norm of $|\nabla u|$, i.e., $\Vert \nabla u\Vert _2^2$. Tikhonov regularization has the strong ability of noise removing. However, it often overly smoothes the image edges. Rudin, Osher and Fatemi in 1992^8^ proposed total variation (TV) regularization to address this over-smoothing problem. The function measured by TV allows for discontinuities along curves during the functional minimization, therefore edges and contours can be preserved in the restoration *u*. Later on, many scholars have done a lot of research on TV regularization, and proposed lots of improved TV-based regularization terms, such as high-order TV^9,10^, hybrid TV^11,12^, non-local TV^13,14^, overlapping TV^15,16^, anisotropic TV^17^. We note that the TV-based regularizers mentioned above are convex functional.

In the last decades, nonconvex regularization based on sparse priors has attracted much attention and found wide applications. It is based on the observation that signals (also images) usually have the very sparse representation in some transformed domains (such as Fourier transform, cosine transform), or in some dictionaries (such as wavelet dictionary, framelet dictionary, self-adaptive dictionary)^18,19^. It is well known that $l_0$-norm measured by the number of nonzero entries is the exact measurement of the sparsity. However, it is difficult to be solved in the practice. A popular method to attack this problem is to use the $l_1$-norm as a relaxation measurement, which is a convex functional and makes the problem easier to solve. It has been shown that under some assumptions, the regularization problems with such $l_1$ relaxation leads to a near optimal sparse solution. To further encourage the sparsity of the solutions, some nonconvex regularizers are proposed since nonconvex functions are much closer to the $l_0$-norm than convex counterparts^20,21^. Since the seminal work of Geman and Geman in^22^, various nonconvex regularization models have been proposed, such as^23–27^. Although nonconvex optimization problems cannot guarantee the existence and uniqueness of the solution, and will lead to complex numerical calculation, a variety of applications (e.g.,^28–31^) have shown that nonconvex regularization models outperform the convex counterparts, and yield the restorations of high quality with sharp and neat edges. In addition, Nikolova et al.^25,30^ provided a theoretical explanation for this phenomenon.

For the **fidelity term**, one always uses the $l_2$-norm $\Vert f-u\Vert _2^2$ to measure the closeness between the restoration *u* and the observation *f*^9–16^. It is well known that such least-squares fitting using $l_2$-norm yields the mean filtering, which is only suitable for removing the additive Gaussian noise, but fails for salt and pepper noise. While the least-absolute fitting using $l_1$-norm leads to the median filtering that is less sensitive to the outliers. So $l_1$-norm fidelity term $\Vert f-u\Vert _1$ is suitable for removing the salt and pepper noise. A lot of regularization models with $l_1$-norm fidelity have been proposed for salt and pepper noise removal, such as^3,26,32–34^. In addition, Chan and Esedoglu in^34^ demonstrated that TV regularization with $l_1$-norm fidelity term (TVL1) is contrast invariant, as opposed to that with $l_2$-norm fidelity term. However, TVL1 model has limited performance in edge-preserving due to the use of the convex TV regularizer. We note in passing that Meyer in^35^ suggested to use some weaker-than-$l_2(\text{also}\; l_1)$ norms as the fidelity term to measure the residual. He introduced three functional spaces, *G*, *E* and *F*, to model the oscillatory functions, which are very suitable for image cartoon-texture decomposition, but not suitable for salt and pepper noise removing.

Based on the above analysis, we note that: (1) TV regularization with $l_1$ fidelity can successfully remove salt and pepper noise, but lacks the ability of edge-preserving; (2) Although nonconvex regularization can preserve image edges well, few studies concern on salt and pepper noise removal. In order to solve these problems, and effectively remove salt and pepper noise while better preserving image edges and contours, a nonconvex $\hbox{TV}_q-l_1$ regularization model is proposed in this paper. It utilizes a nonconvex $\hbox{TV}_q$ regularizer defined in TV domain to model the restoration *u*, and employs the $l_1$-norm as the fidelity term for the noise $f-u$. So, the proposed model can remove the salt and pepper noise while preserving image edges and contours due to the combination of nonconvex regularization and $l_1$-norm fidelity term. A first-order algorithm based on the alternating direction method of multipliers (ADMM) combining with MM scheme proximity operator is developed to numerically solve this nonconvex model. In addition, a sufficient condition for the convergence of the proposed algorithm is provided. The main contributions of this work are as follows:

- A nonconvex $\hbox{TV}_q$ regularization variational model with $l_1$-norm fidelity is proposed. Although much research has been done on the nonconvex regularization and $l_1$-norm fidelity term separately, to the best of our knowledge, there are every few studies on the issue of the combination of the nonconvex regularization and $l_1$-norm fidelity. A few recent works can be seen in^3,26,32^. We note that the nonconvex regularizers in these literatures are defined in the image domain itself, or in the coefficient domain on a basis, whereas our regularizer is in TV domain. Compared with models in^3,26,32^, nonconvex TV regularization has superior performance in edge-preserving, we refer the readers in^25,30^ for more details.
- A first-order algorithm based on ADMM combining with proximity operator is introduced for the nonconvex model. In addition, the convergence property of the proposed algorithm is analyzed. We note that for the “nonconvex regularization + $l_1$-norm fidelity” models, the authors in^3,26,32^ also used ADMM framework. But^3^ did not give a convergence analysis. The authors in^26,32^ derived a convergent algorithm by smoothing the $l_1$-norm fidelity term. Different from the methods in^26,32^, we give a convergence analysis under some nature assumptions to the proposed functional and the parameters.

The structure of this paper is organized as follows. “Introduction” section presents the background and start of this study. “Related work” section gives some background knowledge involving TVL1 model, nonconvex regularization and proximity operator. “Methods” section details the proposed nonconvex $\hbox{TV}_q-l_1$ regularization model, and introduces an efficient numerical algorithm for the proposed model. A sufficient condition for the convergence of the proposed algorithm is also provided in this section. “Results” section discusses the performance of the proposed model and algorithm. “Conclusion” section presents the results. The work ends with concluding remarks.

## Related work

In this section, we recall some background knowledge that are very related to our present work, where TVL1 model is the seminal work for salt and pepper noise removal under the variational regularization framework, nonconvex regularization provides the design of regularizer for the proposed model in this paper, and proximity operator is used to solve the nonconvex subproblem in the ADMM algorithm.

### TVL1 model

Rudin et al.^8^ proposed the following TV regularization model with $l_2$-norm fidelity term (TVL2) to address over-smoothing problem often arising in the Tikhonov regularization,

2 $$\begin{aligned} \mathop {\min }\limits _{u}\left\{ \left\| \nabla u\right\| _1 +\frac{\lambda }{2}\left\| f-u\right\| _2^2\right\} , \end{aligned}$$

where $\left\| \nabla u\right\| _1 = \int _\Omega \left| \nabla u\right| dx$ is the regularization term, and $\Vert f-u\Vert ^2_2$ is $l_2$-norm fidelity term. Model (2) is convex with respect to *u* and easy to be solved in the practice. TVL2 model (2) is suitable for Gaussian noise removing. In addition, TV energy does not penalize the discontinuity of the functions along the contours, so the edges can be preserved in the restoration *u* by model (2).

Chan and Esedoglu in^34^ proposed the following TV regularization model with $l_1$-norm fidelity term (TVL1),

3 $$\begin{aligned} \mathop {\min }\limits _{u}\left\{ \left\| \nabla u\right\| _1 +{\lambda }\left\| f-u\right\| _1\right\} . \end{aligned}$$

TVL1 model (3) is more suitable for salt and pepper noise removing than TVL2 model (2). In addition, compared to TVL2, TVL1 model is contrast invariant. The authors in^34^ gave a simple but illustrative example to show the characteristics of the solutions of TVL1 model and TVL2 model. Assuming the observed image *f*(*x*) being a characteristic function $\mathbf{1 }_{B_r(0)}(x)$ of a disk $B_r(0)$ that is centered at the origin and with radius *r*, they derived the close-form solutions of (2) and (3). The solution of TVL2 model (2) can be written as:

4 $$\begin{aligned} u_{\text{TVL2}}(x) = \left\{ \begin{array}{ll} 0,&{} \quad \hbox{if}\quad 0\; \leqslant \lambda \leqslant 2/r, \\ \left( {1 - \frac{1}{{\lambda r}}} \right) {\mathbf{1 }_{{B_r}(0)}}(x),&{} \quad \hbox{if}\quad \lambda > 2/r. \\ \end{array}\right. \end{aligned}$$

Assuming the minimizer of TVL1 model (3) has to be of the form $c{\mathbf{1 }_{{B_r}(0)}}(x)$ for some constant $c\in [0,1]$, they get the solution as:

5 $$\begin{aligned} {u_{{\text{TVL1}}}}(x) = \left\{ \begin{array}{ll} 0,&{} \quad \hbox{if}\quad 0\; \leqslant \lambda \leqslant 2/r, \\ c{\mathbf{1 }_{{B_r}(0)}}(x),&{} \quad \hbox{if}\quad \lambda = 2/r, \\ {\mathbf{1 }_{{B_r}(0)}}(x),&{}\quad \hbox{if}\quad \lambda > 2/r. \\ \end{array}\right. \end{aligned}$$

From (4) and (5), we observe that both disks in the TVL2 and TVL1 solutions vanish if the radiuses are less than $2/\lambda$. But for the disks whose radiuses *r* are greater than $2/\lambda$, TVL1 model preserves these disks intactly, i.e., $u_{\text{TVL1}} = f$, in contrast to the “contrast loss” phenomena in TVL2 model, where the loss is inversely proportional to $\lambda r$. This intuitive example indicates that TV regularization with $l_1$-norm fidelity can better preserve the contrast of the images than that with $l_2$-norm fidelity in the application of image restoration.

### Nonconvex regularization

From the view of sparse-representation, TV energy is actually the $l_1$-norm of the gradient module, which can be seen as a relaxation of the $l_0$-norm that is the accurate measurement of the sparsity. To promote the sparsity of the entries, nonconvex measurement is a good candidate since it approximates the $l_0$-norm more closely than $l_1$-norm. Nikolova et al.^25,30^ proposed the following nonconvex TV regularization model with $l_2$-norm fidelity term, which is called NTVL2 model in the following:

6 $$\begin{aligned} \mathop {\min }\limits _{u}\left\{ \Vert \varphi \left( \left| \nabla u\right| \right) \Vert _1+\frac{\lambda }{2}\left\| f-u\right\| _2^2\right\} , \end{aligned}$$

where $\varphi (t)$ is a nonconvex potential function, and $\Vert \varphi \left( \left| \nabla u\right| \right) \Vert _1 = \int _\Omega \varphi (|\nabla u|) dx$ is the nonconvex regularization term. Since nonconvex function $\varphi (t)$ is closer to the $l_0$-norm than $l_1$-norm, NTVL2 model (6) can obtain the more sparse representation of $|\nabla u|$ than TVL2 model (2). Furthermore, compared to the TVL2 model, NTVL2 model encourages the penalty to the pattern of small variation, while decreases the penalty to the pattern with large variation. So, NTVL2 model has a superior performance in noise-removing and edge-preserving than classical TVL2 model. However, NTVL2 model (2) is only suitable for Gaussian noise removing due to the use of $l_2$-norm fidelity.

To achieve the sparse recovery in the presence of salt and pepper noise, recently some nonconvex regularization models with $l_1$-norm fidelity have been proposed^3,26,32^, called NRL1, which are defined as follows:

7 $$\begin{aligned} \mathop {\min }\limits _{u} \left\{ P(u)+\frac{\lambda }{2}\left\| f-Au\right\| _1\right\} , \end{aligned}$$

where $P(\cdot )$ is a nonconvex function for sparsity promotion. If *A* is a identity matrix, model (7) is to recover the sparse image *u*. If *A* is a sensing matrix accumulated by a basis, model (7) is to recover the image *Au* which has the most sparse representation on this basis. We note that the nonconvex regularizer in (7) is defined in the image domain itself, or in the coefficient domain on a basis. To inherit the advantages of nonconvex TV regularization in image restoration, we propose a generalized nonconvex regularization variational model for salt and pepper noise removal. It utilizes a generalized nonconvex regularizer defined in the TV domain as the priors to model the restorations, and employs the $l_1$-norm as the fidelity term to measure the noises. New model can effectively remove salt and pepper noise due to the use of $l_1$-norm fidelity; and well preserve image edges and contours due to the use of nonconvex TV regularization.

### Proximity operator of $l_q$ function

The proximity operator is a generalized form of the projection operator, often used to solve non-differentiable optimization problems. In this paper, we use it to solve the nonconvex subproblems in the iterative algorithm. For a proper and lower semi-continuous function *P*(*x*), the corresponding proximity operator is defined as^36,37^,

$$\begin{aligned} {\text{prox}}_{P,\rho }(t) = \mathop {\arg \min }\limits _x \left\{ P(x) + \frac{\rho }{2}\Vert x-t\Vert _2^2\right\} . \end{aligned}$$

Intuitively, proximity operator ${\text{prox}}_{P,\rho }(t)$ is to approximate the point *t* with some other point *x* under the norm $\Vert x-t\Vert _2^2$ and the penalty *P*(*x*). The positive parameter $\rho > 0$ is introduced as a means to control the approximation. In the following, we review the proximity operator for $l_q (0<q<1)$ function, which will be used in our numerical implementation.

When the penalty is given as $l_q$-norm $(0<q<1)$, i.e.,

$$\begin{aligned} P(x)= |x|^q,\;(0<q<1), \end{aligned}$$

the proximity operator does not has a closed-form expression except for two special cases of $q=1/2$ and $q=2/3$. When $P(x)= |x|^{1/2}$, the corresponding proximity operator is a $l_{1/2}$ thresholding function^38,39^,

8 $$\begin{aligned} \hbox{prox}_{P,\rho }(t) = \left\{ \begin{array}{ll} \frac{2}{3}|t|\left( {1 + \cos \left( {\frac{{2\pi }}{3} - \frac{{2{\varphi _\rho }(t)}}{3}} \right) } \right) ,&{}\quad \hbox{if}\quad t > P(\rho ), \\ 0,&{} \quad \hbox{if}\quad |t| \le P(\rho ), \\ - \frac{2}{3}|t|\left( {1 + \cos \left( {\frac{{2\pi }}{3} - \frac{{2{\varphi _\rho }(t)}}{3}} \right) } \right) , &{} \quad \hbox{if}\quad t < - P(\rho ), \\ \end{array} \right. \end{aligned}$$

where

$$\begin{aligned} {\varphi _\rho }(t) = \arccos \left( {\frac{\rho }{8}{{\left( {\frac{{|t|}}{3}} \right) }^{ - \frac{3}{2}}}} \right) , P(\rho ) = \frac{{\root 3 \of {{54}}}}{4}{\rho ^{\frac{2}{3}}}. \end{aligned}$$

When $P(x)= |x|^{2/3}$, the corresponding proximity operator is a $l_{2/3}$ thresholding function^39,40^,

9 $$\begin{aligned} \hbox{prox}_{P,\rho }(t) = \left\{ \begin{array}{ll} \left( {\frac{{|A| + \sqrt{\frac{{2|t|}}{{|A|}} - |A{|^2}} }}{2}} \right) ^3, &{}\quad \hbox{if}\quad t > P(\rho ), \\ 0, &{}\quad \hbox{if}\quad |t| \le P(\rho ), \\ - \left( {\frac{{|A| + \sqrt{\frac{{2|t|}}{{|A|}} - |A{|^2}} }}{2}} \right) ^3, &{}\quad \hbox{if}\quad t < - P(\rho ), \\ \end{array} \right. \end{aligned}$$

where

$$\begin{aligned} |A| = \frac{2}{{\sqrt{3} }}{\rho ^{\frac{1}{4}}}{\left( {\cosh \left( {\frac{\phi }{3}} \right) } \right) ^{\frac{1}{2}}}\quad \hbox{with}\quad \phi = \hbox{arccosh}\left( {\frac{{27{t^2}}}{{16}}{\rho ^{ - \frac{3}{2}}}} \right) \end{aligned}$$

and

$$\begin{aligned} P(\rho ) = \frac{2}{3}{\left( {3{\rho ^3}} \right) ^{\frac{1}{4}}}. \end{aligned}$$

For any other *q*, the authors in^41^ give a semi-implicit expression of the proximity operator with $l_q$-norm penalty, which is defined as:

10 $$\begin{aligned} \hbox{prox}_{P,\rho }(t) = \left\{ \begin{array}{ll} 0 &{}\quad {\text{if}}\quad \left| t \right| < \tau , \\ \left\{ {0,{\text{sign}}(t)\beta } \right\} &{} \quad {\text{if}}\quad \left| t \right| = \tau , \\ {\text{sign}}(t){y^*} &{}\quad {\text{if}}\quad \left| t \right| > \tau . \\ \end{array} \right. \end{aligned}$$

In (10), the threshold $\tau$ satisfies that $\tau = \beta + q \beta ^{q-1}/\rho$ with $\beta = \left( 2(1-q)/\rho \right) ^{\frac{1}{2-q}}$, and $y^*$ is the shrinkage that has not explicit expression. It is a zero point of the non-linear function $h(y)=qy^{q-1}+\rho y- \rho \left| t \right|$ over the region $(\beta ,\;\left| t \right| )$.

## Methods

**Figure 1:**
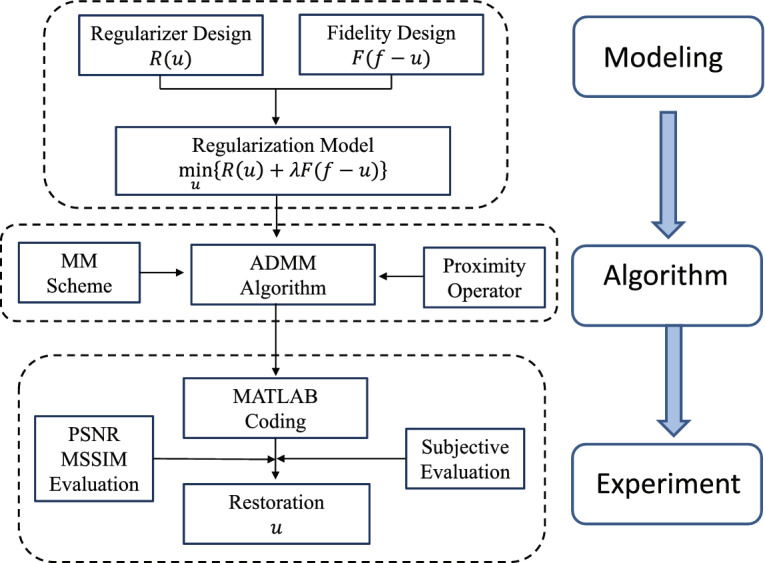
The demonstration of the study design of the present work.

The main purpose of this paper is to effectively remove salt and pepper noise while successfully preserve image edges and contours in image restoration under the variational framework. Firstly, a variational regularization model combining nonconvex regularization and $l_1$ fidelity is proposed, which is actually a minimization problem. And then, the classical ADMM algorithm is developed to numerically solve the proposed model, which is programmed by MATLAB software in the experiments. Finally, some commonly used test images and datasets are used to validate the proposed model and algorithm. The PSNR and MSSIM indexes are used as the means to quantitatively evaluate the performance. Figure 1 shows a flow chart to clarify the study design of the present work.

### The proposed nonconvex $\hbox{TV}_q-l_1$ regularization model

In this section, we present a nonconvex $\hbox{TV}_q-l_1$ regularization variational model, called NTVL1 in the following, which is defined as:

11 $$\begin{aligned} \text{min}\;\;E(u)=P(Bu)+{\lambda }\Vert u-f\Vert _1\quad \text{s.t.}\quad u\in U, \end{aligned}$$

where *P*(*Bu*) is the regularization term, in which $P(\cdot ): {\mathbb {R}}^+ \rightarrow {\mathbb {R}}^+$ is a continuous, increasing and nonconvex $l_q$ function for sparsity promotion, and *B* is the gradient operator $|\nabla |={(\nabla _x^2 + \nabla _y^2)^{1/2}}$. We note in passing that *B* can be choose some other difference operators, such as *x*-directional difference $\nabla _x$, *y*-directional difference $\nabla _y$, and anisotropic difference operator $\nabla _x + \nabla _y$. The $l_1$-norm $\Vert f-u\Vert _1$ is the fidelity term. *U* is a function space (e.g., Sobolev space, bounded variation space). And $\lambda$ is a positive tuning parameter, which balances the regularization term and fidelity term. Model (11) combines the advantages of nonconvex TV regularization and $l_1$-norm fidelity. It can effectively remove the salt and pepper noises by $l_1$-norm fidelity term, while preserving the valuable edges and contours via nonconvex TV regularization.

#### *Remark 1*

Although much research has been done on the nonconvex regularization and $l_1$-norm fidelity term separately, to the best of our knowledge, there are every few studies on the combination of them. A few works can be seen in^26,32^, called NRL1, which are defined as follows:

$$\begin{aligned} \mathop {\min }\limits _{u} \left\{ P(u)+{\lambda }\left\| f-Au\right\| _1\right\} , \end{aligned}$$

where *P*(*u*) is a nonconvex regularization term to measure the sparsity of *u*, and *A* is a transformation matrix. If *A* is a identity matrix, NRL1 model is to recover the sparse image *u*, and if *A* is a sensing matrix accumulated by a basis, it recovers the image *Au* that has the most sparse representation on the basis *A*. But differed from NRL1 regularizer *P*(*u*), our nonconvex regularizer is *P*(*Bu*) that is defined in TV domain. In image restoration application, such scheme can better preserve edges and contours than NRL1.

#### *Remark 2*

The model (11) can effectively preserve edges and contours in the restoration *u* due to the use of nonconvex TV regularization term. Using a basis of a local framework (*N*, *T*), where *N* is normal direction defined as $N=\nabla u /|\nabla u|$, and *T* is the corresponding tangent direction defined as $T=\nabla u^{\perp } /|\nabla u|$, we derive the Euler-Lagrange equation associated with (11),

12 $$\begin{aligned} \frac{P{'}(|B u|)}{|B u|}u_{TT} + P{''}(|B u|)u_{NN} + \lambda \frac{u-f}{|u-f|} = 0 \; \; (|B u|\ne 0 , |u-f|\ne 0), \end{aligned}$$

where $u_{TT}$ and $u_{NN}$ are the second derivatives of *u* in *T* and *N* directions, and $P{'}(|B u|)/|B u|$ and $P{''}(|B u|)$ can be seen as the adaptive diffusion velocity along *T*-direction and *N*-direction, respectively. It is obviously that $P{'}(t)$ is a monotony decrease function and satisfies $P{''}(t)<0$ since *P*(*t*) is a nonconvex increasing function.

Along *T*-direction, for the image pixels where $|B u|\approx 0$ (homogeneous regions), the diffusion Eq. (12) has strong smoothing effect since the diffusivity $P{'}(|B u|)/|B u|$ is of a large value. And for the image pixels where the value of |*Bu*| is large (edges), the model (12) has weak smoothing effect since the value of diffusivity $P{'}(|B u|)/|B u|$ is small. Along *N*-direction, the adaptive diffusivity always satisfies $P{''}(|B u|)<0$ for each image pixel, which means that diffusion in normal direction is always reverse. Based above, we can conclude that the proposed model can effectively smooth the image homogeneous regions, while still preserving the edges and contours very well.

### The proposed algorithm

Obviously, model (11) is a nonconvex and nonsmooth optimization problem since the first term is nonconvex, and the second term is nonsmooth. In this section, we propose an efficient first-order algorithm to solve this model using ADMM framework. ADMM algorithm decouples the variables and makes the global problem easy to tackle, which is very suitable to solve the distributed optimization and high-dimensional optimization problems. With the use of an auxiliary $B u = v$, ADMM algorithm is to solve the following linearly constrained reformulation of (11):

13 $$\begin{aligned} \underset{u,v}{\mathop {\text{min}}}\,\left\{ P(v)+{\lambda }\Vert u-f\Vert _1\right\} \quad \text{s.t.}\quad B u = v,\quad u\in U. \end{aligned}$$

Transforming (13) into an augmented Lagrangian formulation, we obtain

14 $$\begin{aligned} L(u,v;p) = P(v)+{\lambda }\Vert u-f\Vert _1 + \langle p, B u - v\rangle + \frac{\rho }{2}\Vert B u - v\Vert _2^2. \end{aligned}$$

where *u* and *v* are primal variables, *p* is the Lagrangian multiplier, also called dual variable, and $\rho > 0$ is a penalty parameter. Functional (14) can be simplified as:

15 $$\begin{aligned} L(u,v;p) = P(v)+{\lambda }\Vert u-f\Vert _1 + \frac{\rho }{2}\left\| B u - v + \frac{p}{\rho }\right\| _2^2 + C, \end{aligned}$$

where $C = \frac{1}{2\rho }\Vert p\Vert _{2}^2$ that can be neglected in the minimization problem. Then, we alternatively minimize (15) with respect to *u* and *v*, and then update the multiplier *p*. Specifically, the minimization solutions $(u^{k+1}, v^{k+1})$ are obtained alternatively while the other variables are fixed, which leads to the following iteration scheme.

- Step 1. Fixing variables *v* and *p*, we minimize the energy *L*(*u*, *v*; *p*) with respect to *u*.
  16 $$\begin{aligned} {u^{k + 1}} = \mathop {\arg \min }\limits _u \left\{ {\lambda }\Vert u-f\Vert _1 + \frac{\rho }{2}\left\| B u - v^{k} + \frac{p^k}{\rho }\right\| _2^2 \right\} . \end{aligned}$$
- Step 2. Fixing variables *u* and *p*, we minimize the energy *L*(*u*, *v*; *p*) with respect to *v*.
  17 $$\begin{aligned} {{v}^{k + 1}} = \mathop {\arg \min }\limits _v \left\{ P(v) + \frac{\rho }{2}\left\| B u^{k+1} - v + \frac{p^k}{\rho }\right\| _2^2 \right\} . \end{aligned}$$
- Step 3. Updating Lagrangian multiplier *p* as follows:
  18 $$\begin{aligned} {{p}^{k + 1}} = {{p}^k} + \rho \left( B{u^{k + 1}} - {{v}^{k + 1}}\right) . \end{aligned}$$
  ADMM algorithm solves the original model (11) by alternatively updating the above steps. In the following, we solve the subproblems (16) and (17) in detail.

#### Solve the subproblem (16) with respect to *u*

Using an auxiliary variable $w=u-f$, we convert the minimization problem (16) into an equivalent form,

19 $$\begin{aligned} {w^{k + 1}} = \mathop {\arg \min }\limits _w \left\{ F(w) = {\lambda }\Vert w\Vert _1 + \frac{\rho }{2}\left\| B w + Bf - v^{k} + \frac{p^k}{\rho }\right\| _2^2 \right\} . \end{aligned}$$

Then, the optimal $u^{k + 1}$ can be computed by $u^{k + 1} = f + w^{k + 1}$. The *w*-subproblem (19) is actually $l_1$-regularized least squares problem. We use a majorization-minimization (MM) scheme to solve this subproblem approximately. Specifically, let $n^k = v^{k} - Bf - \frac{p^k}{\rho }$, we majorize the quadratic functional $\Vert B w - n^k \Vert _2^2$ in the objective functional (19) with a simple surrogate functional by linearizing it at point $w^k$,

$$\begin{aligned} \left\| {Bw - {n^k}} \right\| _2^2\approx & {} \left\| {B{w^k} - {n^k}} \right\| _2^2 + \left\langle {d\left( {{w^k}} \right) ,\;w - {w^k}} \right\rangle + \frac{1}{{2\tau }}\left\| {w - {w^k}} \right\| _2^2\\= & {} \left\| {B{w^k} - {n^k}} \right\| _2^2 + \frac{1}{{2\tau }}\left\| {w - {w^k} + \tau d\left( {{w^k}} \right) } \right\| _2^2 - \frac{\tau }{2}\left\| {d\left( {{w^k}} \right) } \right\| _2^2, \end{aligned}$$

where $d\left( {{w^k}} \right)$ is the gradient of the quadratic functional $\Vert B w - n^k \Vert _2^2$ at point ${w^k}$, computed by $d\left( {{w^k}} \right) = B^T({Bw^k - {n^k}})$, and $\tau >0$ is a proximal parameter. Using such an approximation of $\left\| {Bw - {n^k}} \right\| _2^2$ in (19), we denote the new energy as $F(w, w^k)$. Obviously, when the proximal parameter $\tau$ satisfies $1/{\tau } > \lambda _{\max }\left( B^T B\right)$, where $\lambda _{\max }\left( B^T B\right)$ denotes the maximum eigenvalue of the matrix $B^T B$, the new energy $F(w, w^k)$ satisfies the classical MM conditions: (i) $F(w, w^k)\ge F({w})$ for all *w*, and (ii) $F(w^k, w^k)= F({w^k})$. Minimizing the surrogate energy $F(w, w^k)$ in stead of the original energy *F*(*w*), and neglecting the constant in the $F(w, w^k)$, we obtain the following minimization problem,

20 $$\begin{aligned} {w^{k + 1}} = \mathop {\arg \min }\limits _w \left\{ {\lambda }\Vert w\Vert _1 + \frac{\rho }{4\tau } \left\| {w - {w^k} + \tau d\left( {{w^k}} \right) } \right\| _2^2 \right\} . \end{aligned}$$

Model (20) is a classical $l_1+l_2$ minimization problem, which can be explicitly solved by a soft thresholding with the shrink operator, i.e.,

21 $$\begin{aligned} {{w}^{k + 1}} = {\text{shrink}}\left( {w^k} - \tau d\left( {{w^k}} \right) ,\;\frac{4\tau \lambda }{\rho } \right) , \end{aligned}$$

where shrink operator is defined as:

$$\begin{aligned} \text{shrink}\left( t, \alpha \right) = \text{sign}(t)\max \{{|t|-\alpha ,0}\}. \end{aligned}$$

#### Solve the subproblem (17) with respect to *v*

Let $m^k = B u^{k+1} + \frac{p^k}{\rho }$, the *v*-subproblem (17) can be computed by the proximity operator, i.e.,

22 $$\begin{aligned} {v}^{k + 1} = \text{prox}_{P,\rho }(m^k), \end{aligned}$$

where $\text{prox}_{P,\rho }$ is the proximity operator for the function $P(\cdot )$ with penalty $\rho$.

The above states the algorithm that solves the augmented Lagrangian formulation *L*(*u*, *v*; *p*) with a fixed *k*. At last, in order to incorporate the algorithm into ADMM framework to solve the original nonconvex model (11), starting with the initial assignment as $k = 0$, ${u^0} = f$, ${v^0} = Bf$ and ${p^0} = 0$, we reiterate the above computing processes, each time updating the value of *k* as $k+1$. Consequently, the ADMM algorithm to our nonconvex variational model (11) is written as follows (Algorithm 1). 
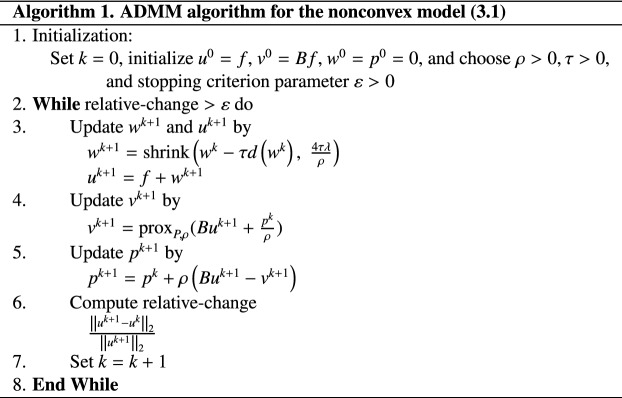


We note that for the Algorithm 1, it only needs one loop to iteratively update the function values. The computation load in each iteration is matrix multiplication. So the complexity of the Algorithm 1 is *O*(*mn*), where *m* is the size of the input images, and *n* is the number of the loop iterations.

### Convergence analysis

In this subsection, we analyze the convergence property of the Algorithm 1. Note that the convergence issue of ADMM algorithm for the convex models has been well addressed, such as^42–45^, while there are very few studies on the nonconvex cases. Inspired by the approaches and conclusions in^46,47^, we derive the following results for convergence of the Algorithm 1. Firstly, several assumptions are introduced, which will be used in the following convergence analysis.

#### **Assumption 1**

Function $P(\cdot )$ is closed, proper and lower semicontinuous.

#### **Assumption 2**

The gradient of $P(\cdot )$ is Lipschitz continuous, i.e., for any *x* and *y*, there exists a positive constant $K>0$, such that:

$$\begin{aligned} {\left\| {\nabla P(x) - \nabla P(y)} \right\| _2} \leqslant K{\left\| {x - y} \right\| _2.} \end{aligned}$$

We note that here we use gradient $\nabla$ rather than derivative since gradient is a generalization of the derivative.

#### **Assumption 3**

The penalty parameter $\rho$ is chosen large enough such that $\rho > K$. In this case, the *v*-subproblem (17) is strongly convex.

#### **Assumption 4**

The energy *E*(*u*) is bounded below, i.e., $\underline{E} = \min E(u) > - \infty$.

We first show that the difference of the dual variable *p* in the iteration can be bounded above by that of the primal variable *v*.

#### **Lemma 1**

*Let* $(u^k,v^k;p^k)$ *be the sequence obtained by Algorithm 1, then we have following*:

$$\begin{aligned} \Vert p^{k+1} - p^k\Vert _2 \le K\Vert v^{k+1} - v^k\Vert _2. \end{aligned}$$

#### *Proof*

From the *v* update step (17), we have the following optimality condition:

$$\begin{aligned} \nabla P (v^{k+1}) - \left( p^k + \rho (Bu^{k+1}-v^{k+1}) \right) = 0. \end{aligned}$$

Combining with the dual variable update step (18), i.e.,

$$\begin{aligned} p^{k+1} = p^k + \rho (Bu^{k+1}-v^{k+1}). \end{aligned}$$

We have

23 $$\begin{aligned} p^{k+1} = \nabla P (v^{k+1}). \end{aligned}$$

By the assumption that the gradient of *P* is Lipschitz continuous, we have

$$\begin{aligned} \Vert p^{k+1} - p^k\Vert _2 = \Vert \nabla P (v^{k+1}) - \nabla P (v^{k})\Vert _2 \le K\Vert v^{k+1} - v^k\Vert _2. \end{aligned}$$

The desired result is obtained. $\square$

Next, we show that the augmented Lagrangian function *L*(*u*, *v*; *p*) is monotonically decreasing in the iterative process.

#### **Lemma 2**

*Let* $(u^k,v^k;p^k)$ *be the sequence obtained by Algorithm 1, then we have following*:

$$\begin{aligned} L\left( u^{k+1}, v^k; p^k\right) - L\left( u^{k}, v^k; p^k\right) \le -\frac{\gamma _1}{4}\left\| u^{k+1} - u^k\right\| _2^2, \end{aligned}$$

*where* $$\gamma _1 = \rho \left( 1/\tau - \lambda _{\max }(B^TB)\right)$$.

#### *Proof*

In (19), let

$$\begin{aligned} h(w)= \left\| Bw + Bf - v^k + {p^k}/{\rho }\right\| _2^2. \end{aligned}$$

In *w*-subproblem (20), we actually minimizes the following approximated objective of (19),

$$\begin{aligned} F\left( {w,{w^k}} \right) = \lambda {\left\| w \right\| _1} + \frac{\rho }{2}\left\langle {\nabla h\left( {{w^k}} \right) ,\;w - {w^k}} \right\rangle + \frac{\rho }{{4\tau }}\left\| {w - {w^k}} \right\| _2^2. \end{aligned}$$

Because $w^{k+1}$ is the minimizer of $F\left( {w,{w^k}} \right)$, we have

$$\begin{aligned} F\left( {{w^{k + 1}},{w^k}} \right) \le F\left( {{w^{k}},{w^k}} \right) , \end{aligned}$$

which implies that:

24 $$\begin{aligned} \lambda {\left\| {{w^{k + 1}}} \right\| _1} + \frac{\rho }{2}\left\langle {\nabla h\left( {{w^k}} \right) ,\;{w^{k + 1}} - {w^k}} \right\rangle + \frac{\rho }{{4\tau }}\left\| {{w^{k + 1}} - {w^k}} \right\| _2^2 \leqslant \lambda {\left\| {{w^k}} \right\| _1}. \end{aligned}$$

Further, the Hessian of *h*(*w*) is $\nabla ^{2}h(w) = B^TB$, we deduce that:

25 $$\begin{aligned} h\left( {{w^{k + 1}}} \right)\leqslant & {} h\left( {{w^k}} \right) + \left\langle {\nabla h\left( {{w^k}} \right) ,\;{w^{k + 1}} - {w^k}} \right\rangle \nonumber \\&+\frac{{{\lambda _{\max }}\left( {{B^T}B} \right) }}{2}\left\| {{w^{k + 1}} - {w^k}} \right\| _2^2, \end{aligned}$$

where $\lambda _{\max }\left( B^T B\right)$ denotes the maximum eigenvalue of the matrix $B^T B$. The inequality (24) combining with (25) yields:

26 $$\begin{aligned}&\lambda {\left\| {{w^{k + 1}}} \right\| _1} + \frac{\rho }{2}h\left( {{w^{k + 1}}} \right) \nonumber \\&\quad \leqslant \lambda {\left\| {{w^{k + 1}}} \right\| _1} + \frac{\rho }{2}h\left( {{w^k}} \right) + \frac{\rho }{2}\left\langle {\nabla h\left( {{w^k}} \right) ,\;{w^{k + 1}} - {w^k}} \right\rangle \nonumber \\&\qquad + \frac{{\rho {\lambda _{\max }}\left( {{B^T}B} \right) }}{4}\left\| {{w^{k + 1}} - {w^k}} \right\| _2^2 \nonumber \\&\quad \leqslant \lambda {\left\| {{w^k}} \right\| _1} + \frac{\rho }{2}h\left( {{w^k}} \right) - \frac{\gamma _1 }{4}\left\| {{w^{k + 1}} - {w^k}} \right\| _2^2, \end{aligned}$$

where $\gamma _1 = \rho \left( 1/\tau - \lambda _{\max }(B^TB)\right)$. With the fact that $w=u-f$, we have

$$\begin{aligned} \left\| {{w^{k + 1}} - {w^k}} \right\| _2^2 = \left\| {{u^{k + 1}} - {u^k}} \right\| _2^2, \end{aligned}$$

which together with (26) yields:

$$\begin{aligned} L\left( u^{k+1}, v^k; p^k\right) - L\left( u^{k}, v^k; p^k\right) \le -\frac{\gamma _1}{4}\left\| u^{k+1} - u^k\right\| _2^2. \end{aligned}$$

The desired result is obtained. $\square$

#### **Lemma 3**

*Let* $(u^k,v^k;p^k)$ *be the sequence obtained by Algorithm 1, then we have following:*

$$\begin{aligned} L\left( u^{k+1}, v^{k+1}; p^k\right) - L\left( u^{k+1}, v^k; p^k\right) \le -\frac{\gamma _2}{2}\left\| v^{k+1} - v^k\right\| _2^2, \end{aligned}$$

*where* $\gamma _2$ *is a positive constant associated with* $\rho$.

#### *Proof*

By the assumption, $\rho >K$ implies that $L\left( u^{k+1}, v; p^k\right)$ is strongly convex respect to the variable *v*. So, we can deduce that there must exist a positive constant $\gamma _2(\rho )$ such that:

27 $$\begin{aligned}&L\left( {{u^{k + 1}},{v^k};{p^k}} \right) \geqslant L\left( {{u^{k + 1}},{v^{k + 1}};{p^k}} \right) \nonumber \\&\quad +\left\langle {{\nabla _v}L\left( {{u^{k + 1}},{v^{k + 1}};{p^k}} \right) ,\;{v^k} - {v^{k + 1}}} \right\rangle \nonumber \\&\quad + \frac{{{\gamma _2}(\rho )}}{2}\left\| {{v^k} - {v^{k + 1}}} \right\| _2^2. \end{aligned}$$

Because $v^{k+1}$ is a minimizer of $L\left( {{u^{k + 1}},{v};{p^k}} \right)$, we have

28 $$\begin{aligned} {\nabla _v}L\left( {{u^{k + 1}},{v^{k + 1}};{p^k}} \right) = 0. \end{aligned}$$

It follows from (27) and (28) that:

$$\begin{aligned} L\left( u^{k+1}, v^{k+1}; p^k\right) - L\left( u^{k+1}, v^k; p^k\right) \le -\frac{\gamma _2}{2}\left\| v^{k+1} - v^k\right\| _2^2. \end{aligned}$$

The desired result is obtained. $\square$

#### **Lemma 4**

*Let* $(u^k,v^k;p^k)$ *be the sequence obtained by Algorithm 1, then we have following:*

$$\begin{aligned}&L\left( {{u^{k + 1}},{v^{k + 1}};{p^{k + 1}}} \right) - L\left( {{u^k},{v^k};{p^k}} \right) \leqslant \\&\quad \left( {\frac{{{K^2}}}{\rho } - \frac{{{\gamma _2}}}{2}} \right) \left\| {{v^{k + 1}} - {v^k}} \right\| _2^2 - \frac{{{\gamma _1}}}{4}\left\| {{u^{k + 1}} - {u^k}} \right\| _2^2. \end{aligned}$$

#### *Proof*

We first split the difference of the augmented Lagrangian function by:

29 $$\begin{aligned}&L\left( {{u^{k + 1}},{v^{k + 1}};{p^{k + 1}}} \right) - L\left( {{u^k},{v^k};{p^k}} \right) \nonumber \\&\quad = L\left( {{u^{k + 1}},{v^{k + 1}};{p^{k + 1}}} \right) - L\left( {{u^{k + 1}},{v^{k + 1}};{p^k}} \right) \nonumber \\&\qquad + L\left( {{u^{k + 1}},{v^{k + 1}};{p^k}} \right) - L\left( {{u^k},{v^k};{p^k}} \right) . \end{aligned}$$

The first term in right side of (29) can be computed by:

30 $$\begin{aligned}&L\left( {{u^{k + 1}},{v^{k + 1}};{p^{k + 1}}} \right) - L\left( {{u^{k + 1}},{v^{k + 1}};{p^k}} \right) \nonumber \\&\quad = \left\langle {{p^{k + 1}} - {p^k},\;B{u^{k + 1}} - {v^{k + 1}}} \right\rangle . \end{aligned}$$

By the dual variable update step (18), we have

31 $$\begin{aligned} Bu^{k+1}-v^{k+1} = \frac{1}{\rho }\left( p^{k+1} - p^k\right) . \end{aligned}$$

Equation (30) together with Eq. (31) yields:

32 $$\begin{aligned} L\left( {{u^{k + 1}},{v^{k + 1}};{p^{k + 1}}} \right) - L\left( {{u^{k + 1}},{v^{k + 1}};{p^k}} \right) = \frac{1}{\rho }\left\| {{p^{k + 1}} - {p^k}} \right\| _2^2. \end{aligned}$$

Further, by Lemma 1, Eq. (32) implies that:

33 $$\begin{aligned} L\left( {{u^{k + 1}},{v^{k + 1}};{p^{k + 1}}} \right) - L\left( {{u^{k + 1}},{v^{k + 1}};{p^k}} \right) \leqslant \frac{{{K^2}}}{\rho }\left\| {{v^{k + 1}} - {v^k}} \right\| _2^2. \end{aligned}$$

The second term in right side of (29) can be split as:

$$\begin{aligned}&L\left( {{u^{k + 1}},{v^{k + 1}};{p^k}} \right) - L\left( {{u^k},{v^k};{p^k}} \right) \\&\quad = L\left( {{u^{k + 1}},{v^{k + 1}};{p^k}} \right) - L\left( {{u^{k + 1}},{v^k};{p^k}} \right) \\&\qquad + L\left( {{u^{k + 1}},{v^k};{p^k}} \right) - L\left( {{u^k},{v^k};{p^k}} \right) , \end{aligned}$$

which together with Lemmas 2 and 3 yields:

34 $$\begin{aligned}&L\left( {{u^{k + 1}},{v^{k + 1}};{p^k}} \right) - L\left( {{u^k},{v^k};{p^k}} \right) \nonumber \\&\quad \leqslant - \frac{{{\gamma _1}}}{4}\left\| {{u^{k + 1}} - {u^k}} \right\| _2^2 - \frac{{{\gamma _2}}}{2}\left\| {{v^{k + 1}} - {v^k}} \right\| _2^2. \end{aligned}$$

Combining Eqs. (33) and (34), we obtain

$$\begin{aligned}&L\left( {{u^{k + 1}},{v^{k + 1}};{p^{k + 1}}} \right) - L\left( {{u^k},{v^k};{p^k}} \right) \leqslant \\&\quad \left( {\frac{{{K^2}}}{\rho } - \frac{{{\gamma _2}}}{2}} \right) \left\| {{v^{k + 1}} - {v^k}} \right\| _2^2 - \frac{{{\gamma _1}}}{4}\left\| {{u^{k + 1}} - {u^k}} \right\| _2^2. \end{aligned}$$

The desired result is obtained. $\square$

Lemma 4 implies that if the condition $\rho \gamma _2 > 2K^2$ is satisfied, then

$$\begin{aligned} L\left( {{u^{k + 1}},{v^{k + 1}};{p^{k + 1}}} \right) - L\left( {{u^k},{v^k};{p^k}} \right) \leqslant 0, \end{aligned}$$

which implies that the value of the augmented Lagrangian function will always decrease with the iteration progressing. We note that as long as parameter $\gamma _2 \ne 0$, one can always find a suitable $\rho$ large enough such that the condition $\rho \gamma _2>2K^2$ is satisfied, since $\rho \gamma _2$ is monotonically increasing with respect to $\rho$, and $2K^2$ is a constant associated with the function $P(\cdot )$.

#### **Lemma 5**

*Let* $(u^k,v^k;p^k)$ *be the sequence obtained by Algorithm 1, then we have following:*

$$\begin{aligned} \mathop {\lim }\limits _{k \rightarrow \infty } L\left( {{u^k},{v^k};{p^k}} \right) \geqslant \underline{E}, \end{aligned}$$

*where* ${\underline{E}}$ *is the lower bound of* *E*(*u*) *defined in Assumption* 4.

#### *Proof*

The augmented Lagrangian function $L\left( {{u^{k + 1}},{v^{k + 1}};{p^{k + 1}}} \right)$ can be expressed as:

35 $$\begin{aligned}&L\left( {{u^{k + 1}},{v^{k + 1}};{p^{k + 1}}} \right) = P({v^{k + 1}}) + \lambda {\left\| {{u^{k + 1}} - f} \right\| _1} \nonumber \\&\quad + \left\langle {{p^{k + 1}},B{u^{k + 1}} - {v^{k + 1}}} \right\rangle + \frac{\rho }{2}\left\| {B{u^{k + 1}} - {v^{k + 1}}} \right\| _2^2. \end{aligned}$$

By (23), $p^{k+1} = \nabla P(v^{k+1})$, (35) can be rewritten as:

36 $$\begin{aligned}&L\left( {{u^{k + 1}},{v^{k + 1}};{p^{k + 1}}} \right) = P({v^{k + 1}})+ \lambda {\left\| {{u^{k + 1}} - f} \right\| _1} \nonumber \\&\quad + \left\langle {\nabla P({v^{k + 1}}),B{u^{k + 1}} - {v^{k + 1}}} \right\rangle + \frac{\rho }{2}\left\| {B{u^{k + 1}} - {v^{k + 1}}} \right\| _2^2. \end{aligned}$$

With the fact that $P(\cdot )$ is a nonconvex function, we have

37 $$\begin{aligned} P({v^{k + 1}})+ \left\langle {\nabla P({v^{k + 1}}),B{u^{k + 1}} - {v^{k + 1}}} \right\rangle \geqslant P({Bu^{k + 1}}). \end{aligned}$$

Since $\left\| {B{u^{k + 1}} - {v^{k + 1}}} \right\| _2^2 \ge 0$, it follows from (36) and (37) that

38 $$\begin{aligned} L\left( {{u^{k + 1}},{v^{k + 1}};{p^{k + 1}}} \right) \geqslant P({Bu^{k + 1}})+ \lambda {\left\| {{u^{k + 1}} - f} \right\| _1} = E({u^{k + 1}}) \end{aligned}$$

Clearly, the inequation (38) together with Assumption 4 imply that the augmented Lagrangian function $L\left( {{u^{k + 1}},{v^{k + 1}};{p^{k + 1}}} \right)$ is bounded below. $\square$

Lemma 4 shows that the augmented Lagrangian function $L\left( {{u^{k}},{v^{k}};{p^{k}}} \right)$ is monotonically decreasing, and Lemma 5 shows that $L\left( {{u^{k}},{v^{k}};{p^{k}}} \right)$ is bounded below. So, we can conclude that the augmented Lagrangian function $L\left( {{u^{k}},{v^{k}};{p^{k}}} \right)$ is convergent as $k\rightarrow \infty$.

#### **Theorem 1**

*Let* $(u^k,v^k,p^k)$ *be the sequence obtained by Algorithm 1, suppose that* $\rho >K$, $\rho \gamma _2 > 2K^2$ *and* $1/\tau > \lambda _{\text{max}}(B^TB)$, *then we have following*:

(i) $$\lim _{k \rightarrow \infty } \left\| {B{u^{k + 1}} - {v^{k + 1}}} \right\| _2^2 = 0$$.
(ii) *If* *U* *is a compact set, then the sequence* $z^k = (u^k,v^k,p^k)$ *converges a limit point* $z^* = (u^*,v^*,p^*)$. *In addition*, $z^*$ *is a stationary point of the augmented Lagrangian function* *L*(*u*, *v*; *p*).

#### *Proof*

We first prove part (i) of the theorem. By Lemmas 4 and 5, we can conclude that the augmented Lagrangian function $L\left( {{u^{k}},{v^{k}};{p^{k}}} \right)$ is convergent as $k\rightarrow \infty$, which implies that:

39 $$\begin{aligned} \mathop {\lim }\limits _{k \rightarrow \infty } \left( {L({u^{k + 1}},{v^{k + 1}};{p^{k + 1}}) - L({u^k},{v^k};{p^k})} \right) = 0. \end{aligned}$$

By Lemma 4, we have

40 $$\begin{aligned}&L\left( {{u^{k + 1}},{v^{k + 1}};{p^{k + 1}}} \right) - L\left( {{u^k},{v^k};{p^k}} \right) \leqslant \nonumber \\&\quad \left( {\frac{{{K^2}}}{\rho } - \frac{{{\gamma _2}}}{2}} \right) \left\| {{v^{k + 1}} - {v^k}} \right\| _2^2 - \frac{{{\gamma _1}}}{4}\left\| {{u^{k + 1}} - {u^k}} \right\| _2^2. \end{aligned}$$

Since $\rho \gamma _2 > 2K^2$ and $\gamma _1>0$, taking limit for (40), and combining (39), we have

$$\begin{aligned} \mathop {\lim }\limits _{k \rightarrow \infty } \left\| {{u^{k + 1}} - {u^{k}}} \right\| _2^2 = 0;\;\;\mathop {\lim }\limits _{k \rightarrow \infty } \left\| {{v^{k + 1}} - {v^{k}}} \right\| _2^2 = 0. \end{aligned}$$

By Lemma 1, we further obtain

41 $$\begin{aligned} \mathop {\lim }\limits _{k \rightarrow \infty } \left\| {{p^{k + 1}} - {p^k}} \right\| _2^2 \leqslant \;{K^2}\mathop {\lim }\limits _{k \rightarrow \infty } \left\| {{v^{k + 1}} - {v^k}} \right\| _2^2 = 0. \end{aligned}$$

With the fact that ${p^{k + 1}}{\text{ = }}{p^k}{\text{ + }}\rho \left( {B{u^{k + 1}} - {v^{k + 1}}} \right)$, using (41), we have

$$\begin{aligned} \mathop {\lim }\limits _{k \rightarrow \infty } \left\| {{p^{k + 1}} - {p^k}} \right\| _2^2{\text{ = }}\;{\rho ^2}\mathop {\lim }\limits _{k \rightarrow \infty } \left\| {B{u^{k + 1}} - {v^{k + 1}}} \right\| _2^2 = 0, \end{aligned}$$

which implies that:

$$\begin{aligned} \mathop {\lim }\limits _{k \rightarrow \infty } \left\| {B{u^{k + 1}} - {v^{k + 1}}} \right\| _2^2 = 0. \end{aligned}$$

Next, we prove part (ii) of the theorem. We first show that there exists a limit point for the sequence $z^k = (u^k,v^k,p^k)$. Since *U* is a compact set, and $\lim _{k \rightarrow \infty } \left\| {{u^{k + 1}} - {u^{k}}} \right\| _2^2 = 0$, there must exist a convergent subsequence $u^{k_{i_1}}$ of $u^{k}$ such that $u^{k_{i_1}}\rightarrow u^*$. Since *B* is a bounded linear operator, and *U* is a compact set, we can deduce that the map set $BU = \left\{ {v:\;Bu = v,\;u \in U} \right\}$ is also a compact set. With the fact that $\lim _{k \rightarrow \infty } \left\| {B{u^{k}} - {v^{k}}} \right\| _2^2 = 0$ and $\lim _{k \rightarrow \infty } \left\| {{v^{k + 1}} - {v^k}} \right\| _2^2 = 0$, we can deduce that $v_k$ also lies in the compact set, and exists a convergent subsequence $v^{k_{i_2}}$ such that $v^{k_{i_2}}\rightarrow v^*$. Note that $\nabla P(v)$ is Lipschitz continuous, and *BU* is a compact set, we can deduce that $\nabla P(v)(v \in BU)$ is bounded, which implies that $\nabla P(v^k)$ is a bounded sequence. With the fact that $p^k = \nabla P(v^k)$ and $\lim _{k \rightarrow \infty } \left\| {{p^{k + 1}} - {p^k}} \right\| _2^2 = 0$, there must exist a convergent subsequence $p^{k_{i_3}}$ such that $p^{k_{i_3}}\rightarrow p^*$, Selecting the same indexes from $\{k_{i_1}\}$, $\{k_{i_2}\}$ and $\{k_{i_3}\}$, denoted as $\{k_i\}$, we have $z^{k_i}\rightarrow z^* = (u^*,v^*,p^*)$ as $k_i\rightarrow \infty$.

Next, we show that any limit point of the sequence $z^k$ is the a stationary point of the augmented Lagrangian function *L*(*u*, *v*, *p*). By the optimality conditions, the sequence $z^k = (u^k,v^k,p^k)$ satisfies that:

42 $$\begin{aligned} \left\{ \begin{array}{l} 0 = \nabla P\left( {{v^{k + 1}}} \right) - {p^k} - \rho \left( {B{u^{k + 1}} - {v^{k + 1}}} \right) , \\ 0 \in \lambda {\partial _u}{\left\| {{u^{k + 1}} - f} \right\| _1} + \rho {B^T}\left( {B{u^{k + 1}} - {v^{k + 1}}} \right) + {B^T}{p^k}, \\ {p^{k + 1}} = {p^k} + \left( {B{u^{k + 1}} - {v^{k + 1}}} \right) . \\ \end{array}\right. \end{aligned}$$

Since $z^{k_i}\rightarrow z^* = (u^*,v^*,p^*)$ as $k_i\rightarrow \infty$, passing to the limit in (42) along the subsequence $z^{k_i}$, we obtain

$$\begin{aligned} \nabla P\left( {{v^ * }} \right) = {p^*};\;0 \in \lambda {\partial _u}{\left\| {{u^*} - f} \right\| _1} + {B^T}{p^*};\;B{u^*} = {v^*}, \end{aligned}$$

which implies that $z^* = (u^*,v^*,p^*)$ is a stationary point of the augmented Lagrangian function *L*(*u*, *v*, *p*). The desired result is obtained. $\square$

## Results

In this section, we show the effectiveness of the proposed model and algorithm in image denoising application. The programs are coded in MATLAB, and run on a PC with Intel Core i5 2.5G CPU and 4.00G RAM. The peak signal to noise ratio (PSNR) and mean structural similarity (MSSIM) index^48^ are used as the means of judging the performance. The main experimental content of this paper is as follow:

1. The effectiveness of the proposed model, and the convergence of the algorithm.
2. The effect of the nonconvex parameter *q* in the proposed model.
3. The comparison with TVL2, NTVL2, TVL1, TGV, NLTV, NRL1, ASWMF and BM3D models on some test images and datasets.

In all experiments, the difference operator *B* in the model (11) is chosen as the gradient operator $|\nabla |$. Then, $P(|\nabla u|)$ is the nonconvex total variation measure of the input *u*. Here, we give the definition of $\nabla$ in the discrete case. Rearranging the two-dimensional image matrix *u* in (11) into a vector by scanning the column one by one, we define the gradient operator $\nabla$ in a matrix form,

$$\begin{aligned} \nabla = \left[ \begin{array}{l} {\nabla _x} \\ {\nabla _y} \\ \end{array} \right] = \left[ \begin{array}{l} {I_n} \otimes {\nabla _1} \\ {\nabla _1} \otimes {I_n} \\ \end{array} \right] , \end{aligned}$$

where $I_n$ is the *n*-dimensional identity matrix, $\otimes$ denotes the Kronecker product, and $\nabla _1$ is difference elementary matrix defined as:

$$\begin{aligned}{\nabla _1} = \left[ \begin{array}{cccc} 0 &{} {} &{} {} &{} {} \\ -1 &{} 1 &{} {} &{} {} \\ {} &{} \ddots &{} \ddots &{} {} \\ {} &{} {} &{} -1 &{} 1 \\ \end{array} \right] _{n \times n}. \end{aligned}$$

Then, let *u* be an image in ${\mathbb {R}}^{n^2}$. The gradient of *u* can be computed as:

$$\begin{aligned}\nabla u = \left[ \begin{array}{l} {\nabla _x}u \\ {\nabla _y}u \\ \end{array}\right] = \left[ \begin{array}{l} ({I_n} \otimes {\nabla _1}) u \\ ({\nabla _1} \otimes {I_n}) u \\ \end{array}\right] . \end{aligned}$$

In the proposed model (11), we use $l_q$-norm as the nonconvex penalty function, i.e., $P(x)=|x|^q$. Here, we only use the $l_q$-norm penalty since (1) it has a flexible parametric form; (2) it’s proximity operator corresponds to a thresholding function that is easy to compute in the practice; (3) the popular hard- and soft-thresholding is the special cases of our $l_q$ thresholding. By Theorem 6, the parameter $\rho$ must be chosen large enough to guarantee the convergence conditions. However, the ADMM algorithm would be every slow and impractical if with a very large value of $\rho$. In this paper, we adopt the scheme in^26^ to address this problem. Starting with a properly small value of $\rho$, we gradually increase the values of $\rho$ in the iteration until reaching the target value, i.e., $0<\rho _0<\rho _1<\cdots <\rho _k\cdots$. The stopping criterion for the proposed algorithm is that the relative-change between the restored images of the successive iterations is smaller than $\varepsilon = 10^{-3}$. The parameter $\tau$ is set as $\tau = 0.9/\lambda _{\text{max}}(B^TB)$; $\lambda$ is manually tuned such that the restoration achieves the largest PSNR value.

### The effectiveness of the proposed model

**Figure 2:**
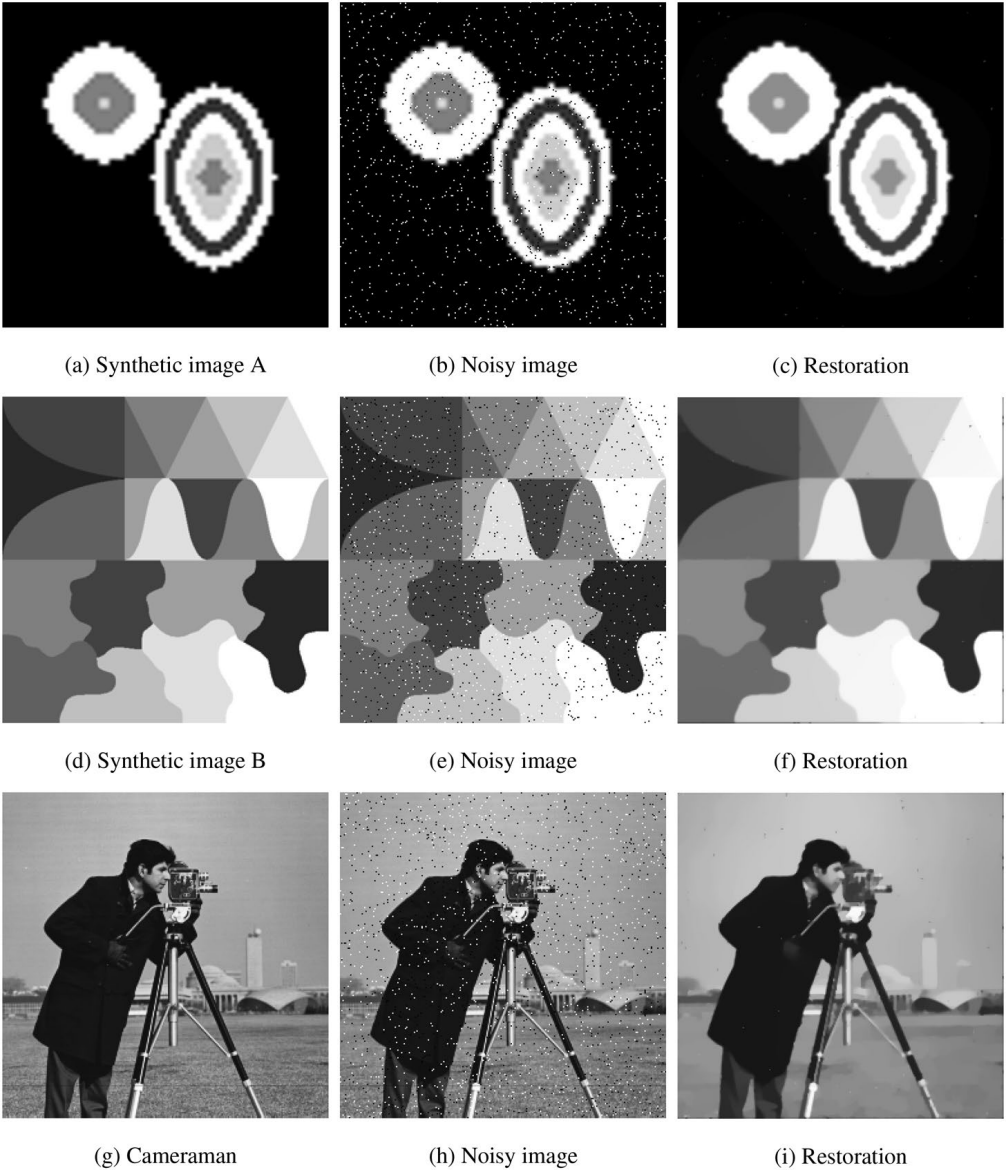
The denoising results of the proposed model.

The first experiment aims to show the effectiveness of the proposed model and algorithm in image denoising application. The nonconvex regularization function is chosen as $P(x)=|x|^{1/2}$ whose corresponding proximity operator is computed by (9). Test images shown in the first column of Fig. 2 are two synthetic images and two real images with the size of $256\times 256$. The second column of Fig. 1 shows the corresponding noisy versions obtained by adding the salt and pepper noises with the density of 0.03 into the clean data. Here, Matlab built-in function $imnoise$ are used to contaminate the images. The denoising results are shown in the last column of Fig. 1. From the results, we observe the following: (1) The proposed model is very effective for salt and pepper noises removing due to the use of the $l^1$ fidelity term. Almost all salt and pepper noises are removed in the restorations; (2)The image edges and contours can be preserved well by using nonconvex TV regularization.

**Figure 3:**
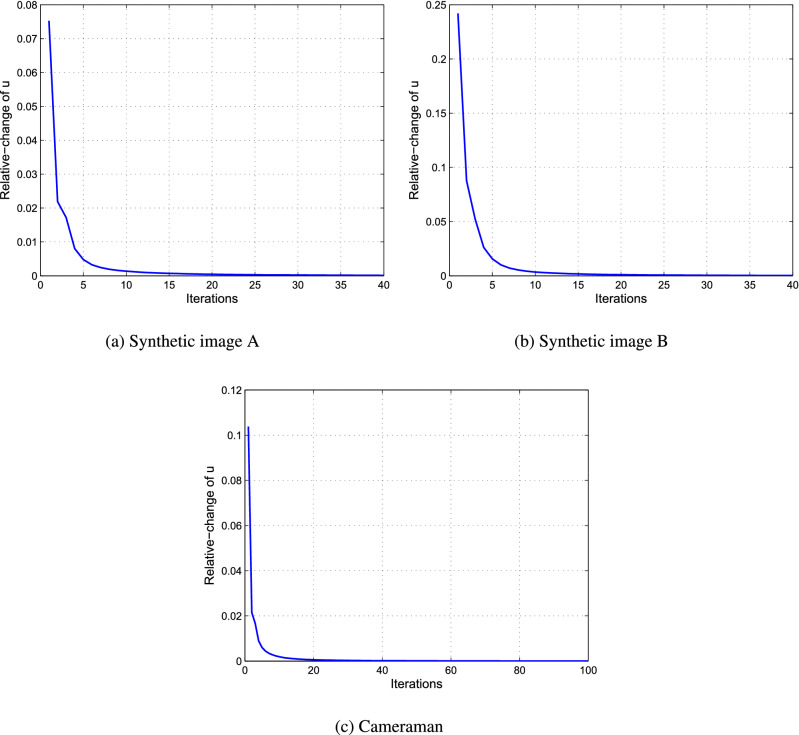
The plots of the relative-change of the restorations versus iterations.

**Figure 4:**
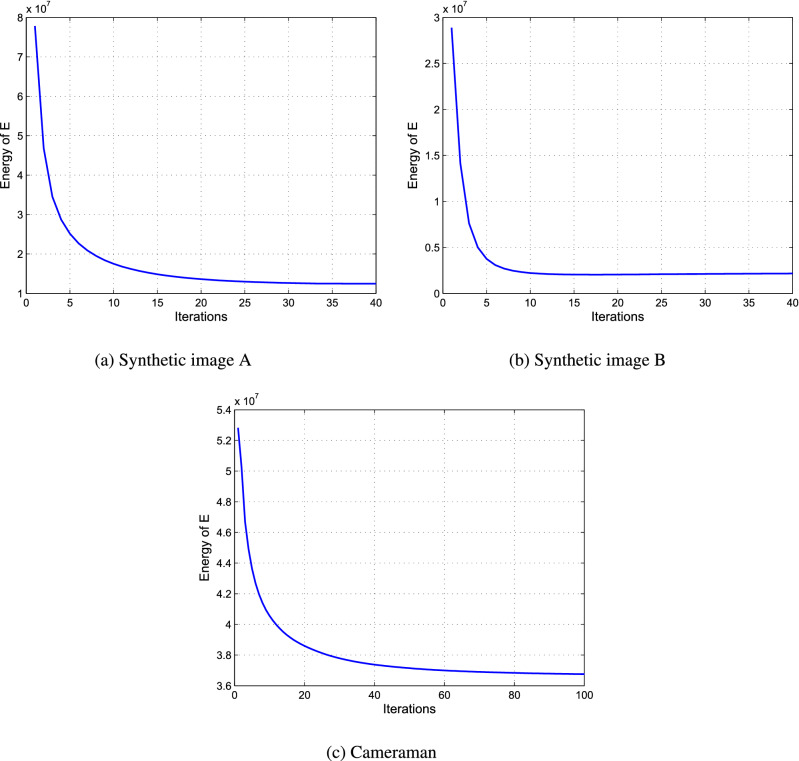
The plots of the energy *E* versus iterations.

Next, we demonstrate the convergence property of the proposed algorithm by plotting two measures of the sequence $u^k$ conducted by Algorithm 1. Here, the test data are the images in the first experiment. Figure 3 shows the plots of the relative-change of the restorations versus iterations, where the relative-change of the restoration *u* in the iteration is computed by ${{\left\| {{u^{k + 1}} - {u^k}} \right\| }_2}/{{{\left\| {{u^{k + 1}}} \right\| }_2}}$. Figure 4 shows the plots of the energy $E(u^k)$ computed by (11) versus iterations. From Fig. 4, we can see that the relative-change of *u* significantly decreases in the first few steps, and then converges to zero, which implies that $u^{k + 1} \rightarrow {u^k}$ as $k\rightarrow \infty$ in the $l^2$ topology. And from Fig. 4, we observe that the energy *E* firstly decreases with the iteration progressing, and then converges to a constant, which implies that the limit point of the sequence $u^k$ is a local minimum point of the functional *E*(*u*). These two figures support the convergence analysis of the proposed algorithm in “The comparison experiment” section.

### The test of different nonconvex parameter *q*

In this section, we test the $l_q$-norm nonconvex penalty functions with different *q*-values in the interval of (0, 1). In the proposed algorithm, the *v*-subproblem is updated by proximity operator with $l_q$-norm penalty. We note that when $q=1/2$ and $q=2/3$, the corresponding proximity operators are $l_{1/2}$ and $l_{2/3}$ thresholding functions, which can be explicitly computed by (9) and (10), respectively. For any other values of *q*, the corresponding proximity operators are computed by (2.9), in which we need to solve a zero point $y^*$ of the non-linear function $h(y)=qy^{q-1}+\rho y- \rho \left| t \right|$. In the numerical implementation, the zero point $y^*$ is solved by Newton method since *h*(*y*) is a convex function.

**Figure 5:**
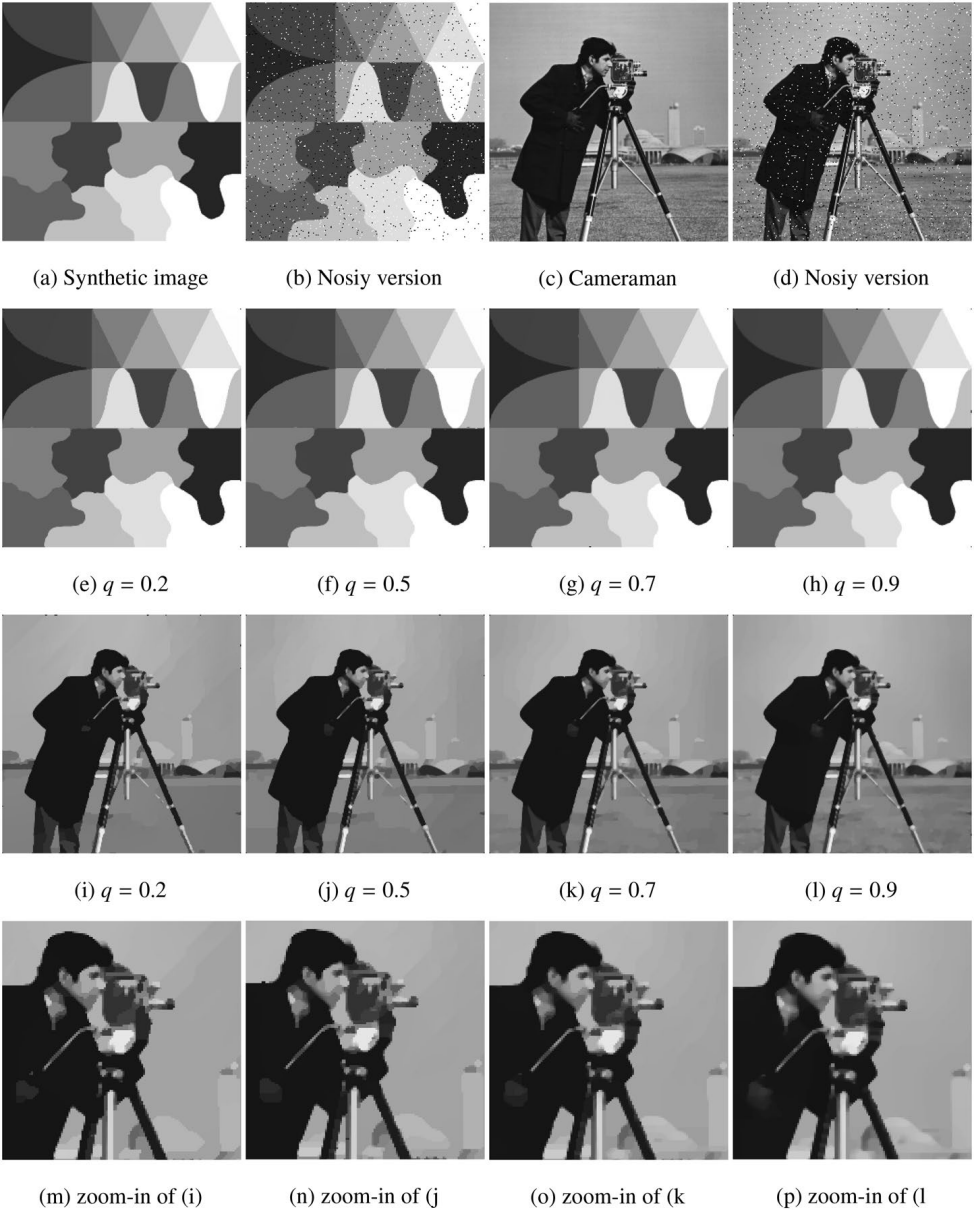
The test of $l_q$-norm nonconvex penalty functions with different *q*-values.

Figure 5 shows the denoising results of the proposed model with different values of *q*, $q \in \{0.2, 0.5, 0.7, 0.9\}$, for two test images (Synthetic image A and Cameraman) with the size of $256 \times 256$, where the the noisy images are obtained by adding the salt and pepper noises with the density of 0.03 into the clean data. We observe that, with these different nonconvex functions, the models all can remove the salt and pepper noises while preserving edges and contours in the restorations. However, the PSNR values listed in Table 1 show that in restoring the synthetic image, $q = 0.2$ yields the best performance, which is different from the results in restoring the Cameraman image, where $q = 0.7$ yields the best performance. In our opinion, this is due to the nature that real images are not strictly sparse as the synthetic sparse images in the TV domain.

**Table 1:** PSNR for NTVL1 model with different penalty functions.

Images	Noisy	$q=0.2$	$q=0.5$	$q=0.7$	$q=0.9$
Synthetic image	19.4521	32.2447	32.1714	31.8729	31.8704
Cameraman	19.7212	29.6341	29.7428	30.1026	29.8905

### The comparison experiment

In this subsection, we compare the proposed model with several state-of-the-art models in denoising application.

#### The comparison with TVL2, NTVL2 and TVL1

**Figure 6:**
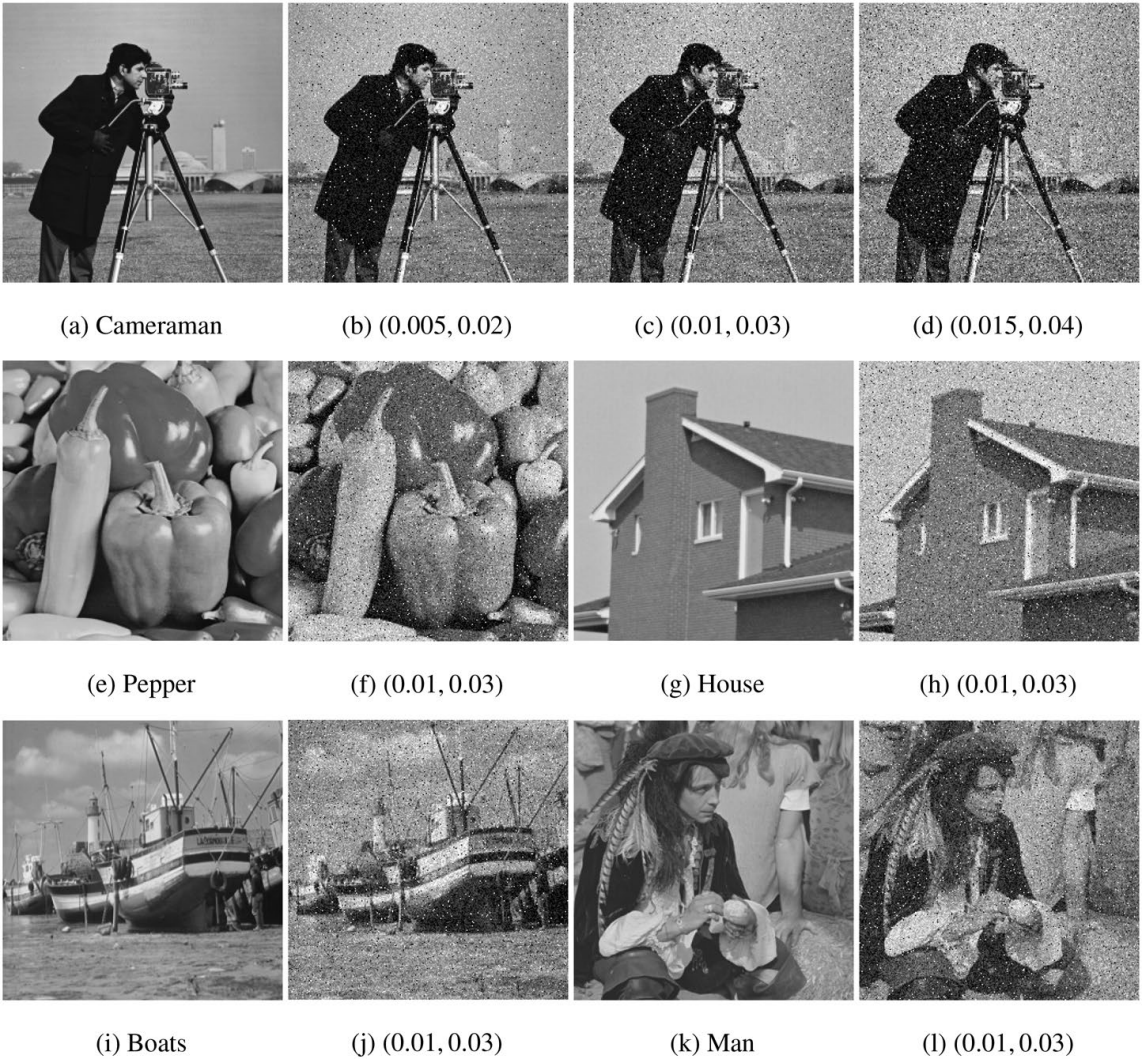
The test images.

**Figure 7:**
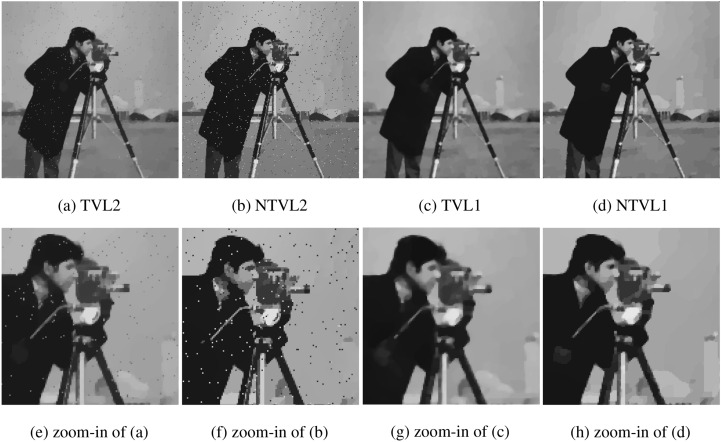
The denoising results of Cameraman contaminated by Gaussian noise ($\sigma = 0.005$) and salt and pepper noise ($d=0.02$).

**Figure 8:**
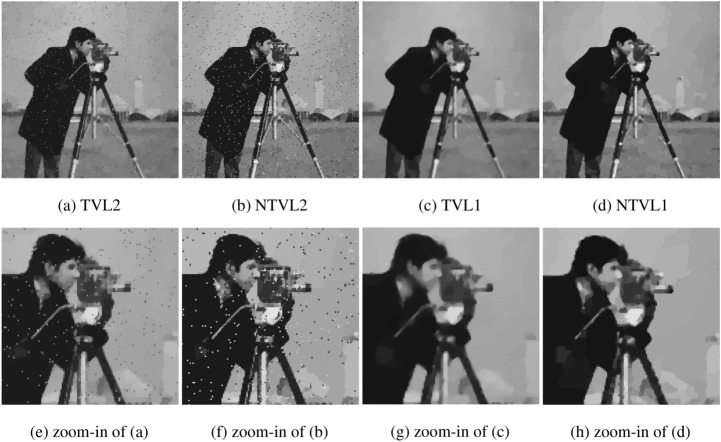
The denoising results of Cameraman contaminated by Gaussian noise ($\sigma = 0.01$) and salt and pepper noise ($d=0.03$).

**Figure 9:**
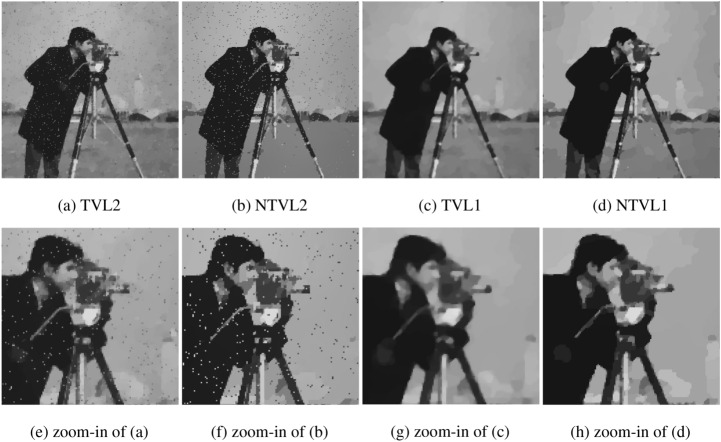
The denoising results of Cameraman contaminated by Gaussian noise ($\sigma = 0.015$) and salt and pepper noise ($d=0.04$).

In this experiment, we compare the proposed model with TVL2^8^, NTVL2^25^ and TVL1^34^ models. Firstly, we denoise Cameraman image that is contaminated by mixed Gaussian noise and salt and pepper noise of different levels. Test images are shows in the first row of Fig. 6, which are obtained by adding Gaussian noise with variance of $\sigma = 0.005, 0.01, 0.015$, mixed with salt and pepper noise with density of $d = 0.02, 0.03, 0.04$ to the clean data, respectively. We use Matlab built-in function $imnoise$ twice to add the mixed noises. The PSNR and MSSIM values of the noisy versions are listed in Table 2. The restoration results of the noisy Cameraman with different noise levels are shown in Figs. 7, 8 and 9, respectively. In these figures, the first row shows the denoising results using the four models, and the second row shows the corresponding local zoomed-in regions of the restorations. The PSNR and MSSIM values for all restorations are listed in Table 3. From the numerical results, we have the following conclusions:

- $l_1$-norm fidelity term is more effective for salt and pepper noise and outlier removing than $l_2$-norm. We observe that TVL2 and NTVL2 models perform well for Gaussian noise removing, but fail for salt and pepper noise. Some impulsive points are still remained in the restorations obtained by TVL2 and NTVL2 models. For TVL1 and NTVL1 models, however, it can be seen that these two models can remove the salt and pepper noise successfully. Almost all impulsive points are removed from the restorations by TVL1 and NTVL1 models.
- Nonconvex regularization has the better performance in edges and contours preserving than convex ones. Comparing the restorations of TVL2 and NTVL2 models, we observe that NTVL2 model keeps sharp features better than TVL2 model, even those impulsive points that are more prominent in the restorations by NTVL2 model. NTVL1 and TVL1 models can remove the impulsive points due to the use of the $l_1$-norm fidelity. But obviously, the NTVL1 model significantly outperforms TVL1 model in preserving sharp contours and details. For example, in the face and camera of the cameraman, NTVL1 model restores more details and features than TVL1 model. In addition, Table 3 show that restorations by the NTVL1 model have slightly greater PSNR and MSSIM values than TVL1 model, which further demonstrates that nonconvex regularization has the better performance than convex regularization.
- The proposed NTVL1 model has the largest PSNR and MSSIM values within these four models. It indicates that the combination of nonconvex regularization and $l_1$-norm fidelity is promising in restoring the images contaminated by mixed Gaussian noise and salt and pepper noise.

**Table 2:** PSNR and MSSIM for the test images.

Images	Noise level	PSNR	MSSIM
Cameraman	$\sigma =0.005,\;d=0.02$	19.8379	0.7021
Cameraman	$\sigma =0.010,\;d=0.03$	17.5835	0.6251
Cameraman	$\sigma =0.015,\;d=0.04$	16.3325	0.5573
Pepper	$\sigma =0.010,\;d=0.03$	17.7076	0.6285
House	$\sigma =0.010,\;d=0.03$	17.7013	0.6274
Boats	$\sigma =0.010,\;d=0.03$	17.6472	0.6201
Man	$\sigma =0.010,\;d=0.03$	17.7343	0.6342

**Figure 10:**
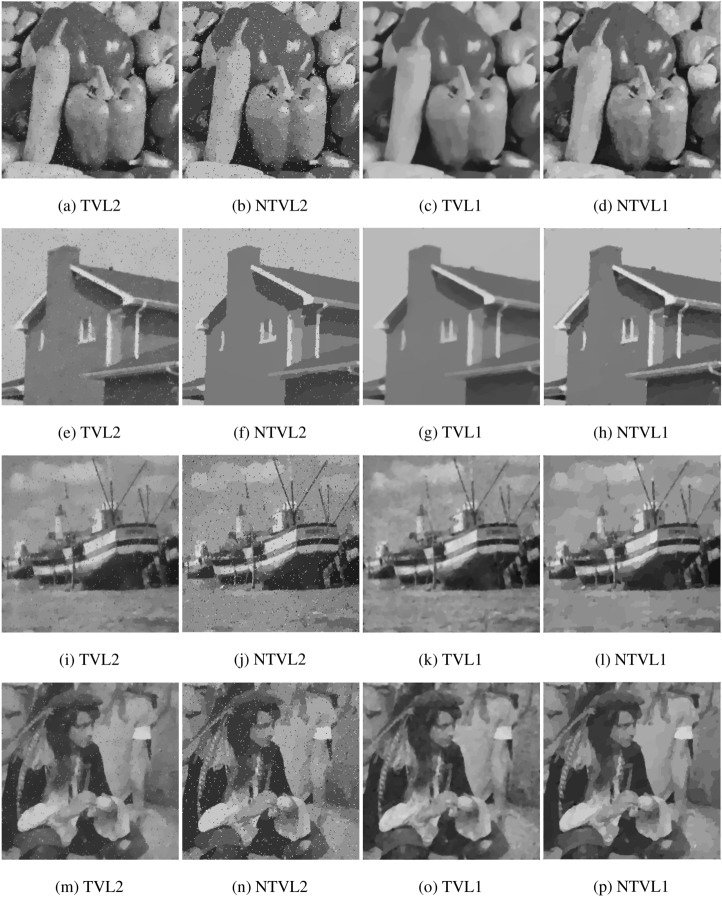
The denoising results of noisy Pepper, House, Boats and Man.

**Figure 11:**
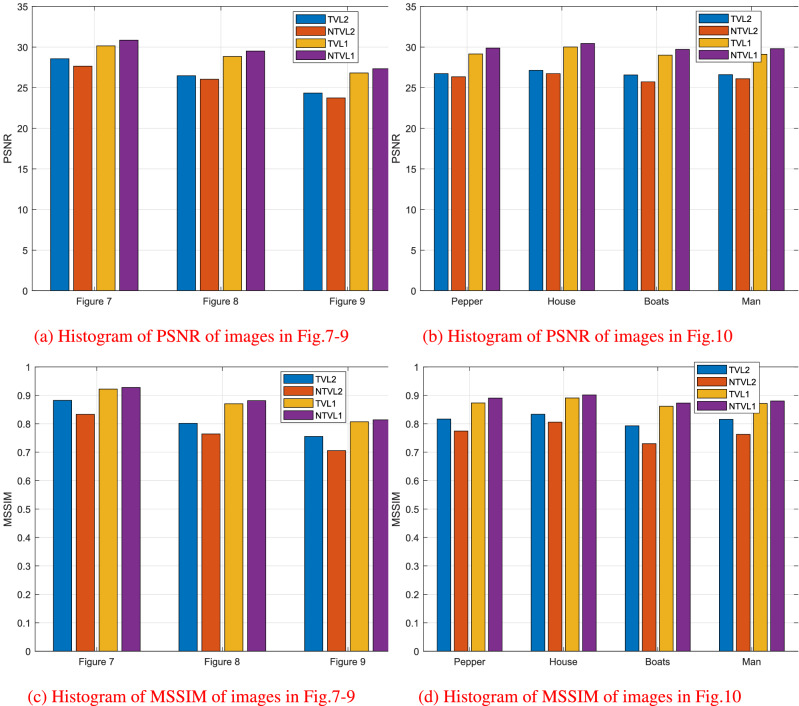
The Histogram of PSNR and MSSIM for different models.

Next, we apply the proposed model to several real images. Test images shown in Fig. 5 are “Pepper”, “House”, “Boats” and “Man” images with the size of $256 \times 256$, which contain lots of edges, contours, details, textures, inhomogeneous regions and features of low contrast and so on. The noisy versions are obtained by adding the mixed Gaussian noise with $\sigma = 0.01$ and salt and pepper noise with $d = 0.03$ to the clean data. The PSNR and MSSIM values of the test noisy images are shown in Table 2. Again, we compare the proposed model with TVL2, NTVL2 and TVL1 models. Figure 10 shows the restoration results. One can clearly see that TVL2 and NTVL2 model can successfully remove Gaussian noise while preserving edges and contours. But they fail to remove salt and pepper noise. Some impulsive points are still remained in the restorations. TVL1 and NTVL1 can simultaneously remove Gaussian noise and salt and pepper noise while preserving the edges. But we can see that the proposed model preserves more image contours and details than TVL1. Table 3 and Figure 11 show that NTVL1 model has the largest PSNR and MSSIM values, which further demonstrates that our model has the best performance in restoring the images contaminated by mixed Gaussian and salt and pepper noise in these four models due to the use of the combination of nonconvex TV regularization and $l_1$-norm fidelity.

**Table 3:** PSNR and MSSIM for TVL2, NTVL2, TVL1 and NTVL1 models.

Images	TVL2		NTVL2		TVL1		NTVL1	
	PSNR	MSSIM	PSNR	MSSIM	PSNR	MSSIM	PSNR	MSSIM
Figure 7	28.5674	0.8823	27.6502	0.8332	30.1432	0.9221	30.8473	0.9276
Figure 8	26.4748	0.8014	26.0423	0.7643	28.8482	0.8704	29.5037	0.8812
Figure 9	24.3374	0.7553	23.7385	0.7054	26.8120	0.8071	27.3345	0.8137
Pepper	26.7285	0.8165	26.3427	0.7745	29.1487	0.8732	29.8821	0.8904
House	27.1347	0.8334	26.7348	0.8057	30.0143	0.8908	30.4465	0.9013
Boats	26.5703	0.7927	25.7250	0.7302	29.0032	0.8616	29.7231	0.8728
Man	26.6031	0.8156	26.1026	0.7627	29.1179	0.8715	29.8032	0.8801

#### The comparison with TGV, NLTV, NRL1, ASWMF and BM3D

In this experiment, we compare the proposed model with very famous total generalized variation (TGV)^10^, nonlocal total variation (NLTV)^13^, adaptive switching weighted median filter (ASWMF)^49^, nonconvex regularization model with $l_1$-nrom fidelity (NRL1)^26^, and block-Matching and 3D filtering (BM3D)^50^. TGV is a high-order variation regularization model, which can well restore piecewise smooth regions while preserving the edges. NLTV uses patch-distance rather than point-distance to measure the nonlocal similarity of the image, which can better restore the image details than classic TV based models. ASWMF is based on median filting which can well remove the salt and pepper noise. NRL1 is a robust sparse recovery model with $l_1$-norm fidelity, which can well restore the sparse image, or image with sparse representation on some basises. In the experiment, as in^26^, the sensing matrix *A* in NRL1 (8) is chosen as a partial discrete cosine transformation matrix. BM3D is a hybrid model, which combines block-Matching, 3D linear transform thresholding, and Wiener filtering. It is probably one of the best methods so far in image denoising application.

**Figure 12:**
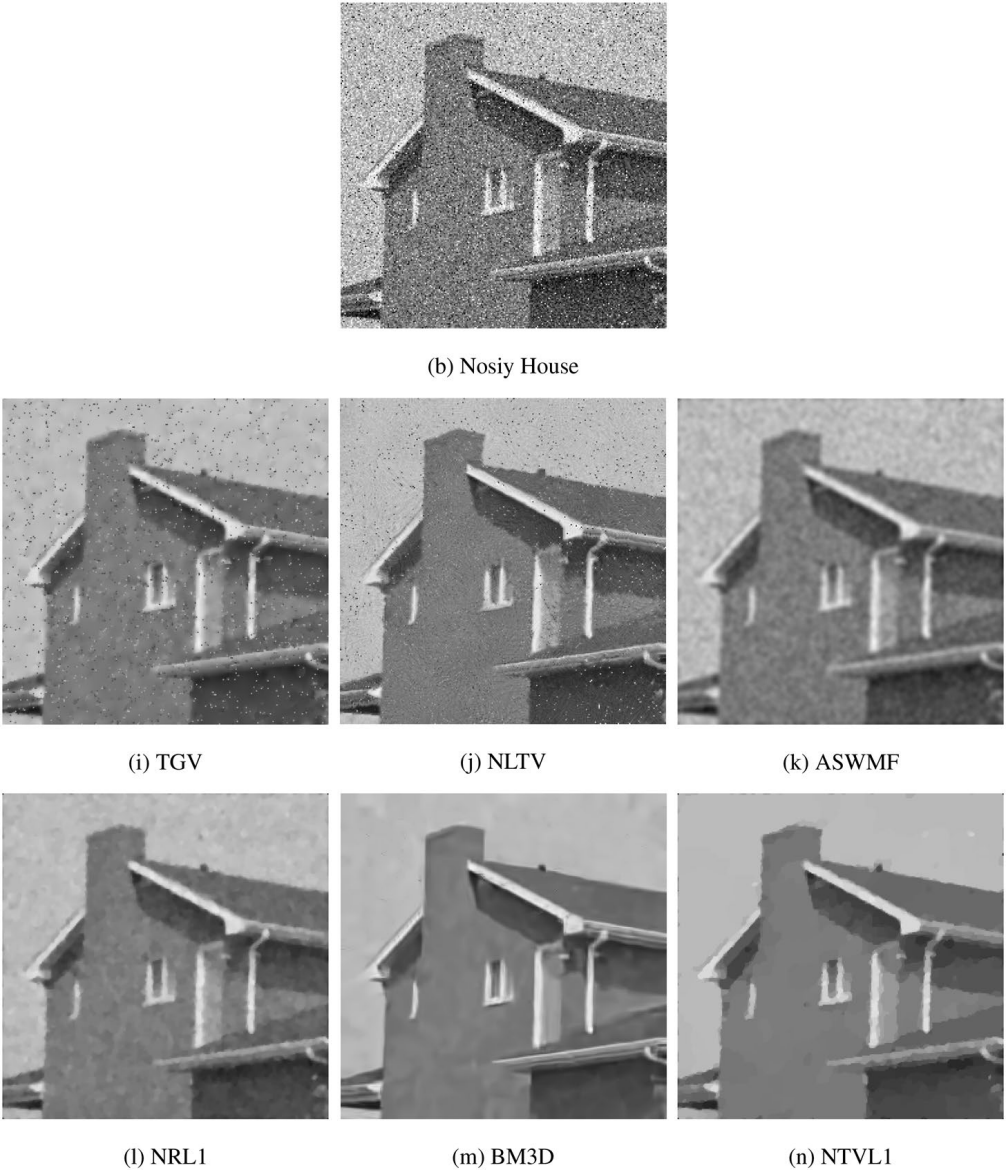
The comparison with TGV, NLTV, NRL1, ASWMF and BM3D.

**Figure 13:**
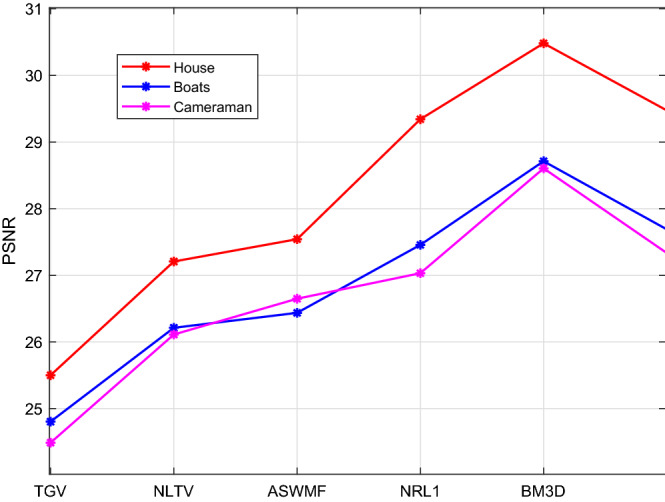
The plots of the relative-change of the restorations versus iterations.

We use three images (“House”, “Boats” and “Cameraman”) with the size of $256\times 256$ as the test data for comparisons. All images are contaminated by mixed Gaussian noise with $\sigma = 0.015$ and salt and pepper noise with $d = 0.04$. The restoration results are shown in Fig. 12. To save space, we here only show the result of House. We can see that TGV and NLTV can remove the Gaussian noise, but fail to remove salt and pepper noise. NLTV obtain the results with higher visual quality than TGV. ASWMF can well remove the salt and pepper noise, but blurs the edges. Our model and NRL1 can successfully remove the mixed noises, while well preserving the images edges and contours. BM3D has the best performance in terms of visual quality. The PSNR values are listed in Table 4 and Fig. 13. From the results, we note that the proposed model obtains the results with higher PSNR values compared with TGV, NLTV, ASWMF and NRL1. BM3D has the largest PSNR values in these six models. Although BM3D works better than the proposed model, we think that the proposed model is still worthy of consideration since it needs lower computational complexity compared to BM3D, and outperforms other popular models.

**Table 4:** The PSNR for TGV, NLTV, ASWMF, NRL1, BM3D and NTVL1 models.

Images	Noisy	TGV	NLTV	ASWMF	NRL1	BM3D	NTVL1
House	16.2501	25.5008	27.2079	27.5425	29.3425	30.4817	29.4744
Boats	16.2793	24.8049	26.2132	26.4373	27.4574	28.7125	27.6892
Cameraman	16.2284	24.4891	26.1124	26.6492	27.0323	28.6056	27.3345

#### The comparison in Set5 and Set13 datasets

**Figure 14:**
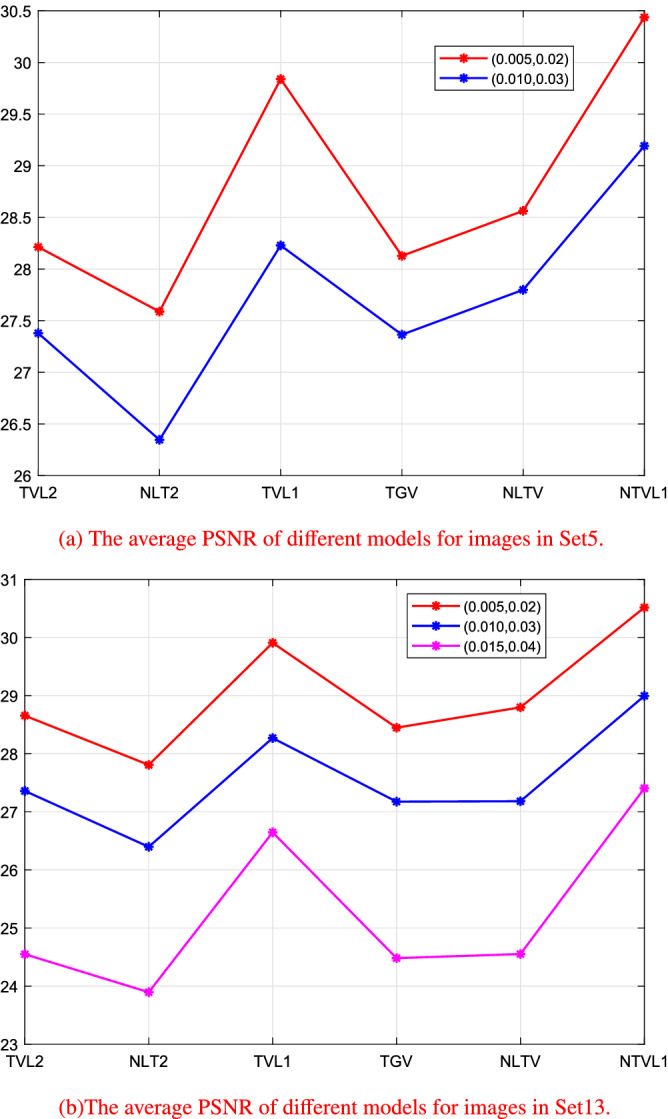
The plots of the relative-change of the restorations versus iterations.

In the last experiment, to further show the effectiveness and adaptability of the proposed model, we test the proposed model on Set5 and Set13 datasets^28^. The test images in these two datasets are contaminated by mixed Gaussian noise and salt and pepper noise. Again, we compare the proposed model with five TV based models: TVL2, NTVL2, TVL1, TGV and NLTV. The PSNR values of the results are shown in Tables 5 and 6. The second column in the tables is the noise level. The two numbers are the variance of the Gaussian noise and the density of the salt and pepper noise, respectively. And Fig. 14 shows the line chart of the average PSNR on the two datasets. From the results, we observe that the proposed model achieves the best performance in terms of PSNR on Set5 and Set13 datasets. It yields about 0.5 dB PSNR and 0.06 MSSIM improvements against all compared models.

**Table 5:** The PSNR of the restored images in Set5.

Images	Noise level	TVL2	NTVL2	TVL1	TGV	NLTV	NTVL1
Baby	$(0.005,\;0.02)$	28.2722	27.7786	30.0166	28.2438	28.4745	30.7418
Bird	$(0.005,\;0.02)$	28.3072	27.5825	29.6969	28.1984	28.7564	30.0999
Butterfly	$(0.005,\;0.02)$	28.0594	27.4015	29.8055	27.9386	28.4578	30.4686
Head	$(0.010,\;0.03)$	27.3612	26.3291	28.3278	27.4926	27.7044	29.2682
Woman	$(0.010,\;0.03)$	27.3959	26.3629	28.1289	27.2359	27.8926	29.1155

**Table 6:** The PSNR of the restored images in Set13.

Images	Noise level	TVL2	NTVL2	TVL1	TGV	NLTV	NTVL1
Baboon	$(0.005,\;0.02)$	28.5925	27.4458	29.7545	28.4815	28.7382	30.3338
Barbara	$(0.005,\;0.02)$	28.6531	28.0490	29.6462	28.4925	28.6292	30.3059
Bridge	$(0.005,\;0.02)$	28.4917	27.7452	29.7116	28.3681	28.5960	30.2850
Coastguard	$(0.005,\;0.02)$	28.8866	27.9923	30.5251	28.4509	29.2313	31.1448
Comic	$(0.010,\;0.03)$	26.7026	25.9285	27.4109	26.3028	26.4793	27.8180
Face	$(0.010,\;0.03)$	27.6718	26.5550	28.6417	27.7066	27.7051	29.6331
Flowers	$(0.010,\;0.03)$	27.2734	26.2021	28.1997	26.9240	26.8204	28.8680
Foreman	$(0.010,\;0.03)$	27.7899	26.9040	28.8294	27.7680	27.7259	29.6630
Man	$(0.015,\;0.04)$	24.5600	23.6500	26.6661	24.5250	24.7719	27.9400
Monarch	$(0.015,\;0.04)$	24.6650	23.7680	26.7085	24.5745	24.5973	27.2110
Pepper	$(0.015,\;0.04)$	24.9870	24.4955	27.2536	24.6818	25.1831	28.1390
Ppt3	$(0.015,\;0.04)$	24.4412	23.8398	26.7101	24.6009	24.5333	26.9494
Zebra	$(0.015,\;0.04)$	24.0921	23.7238	25.8972	24.0275	23.6755	26.7856

Based on the above experiments, we can obtain the following results: (1) The proposed $\hbox{TV}_q-l_1$ model is effective for salt and pepper noise removal while preserving image edges and contours quite well. (2) The convergence of the proposed algorithm is verified by experiments. (3) Compared with TVL2, NTVL2, TVL1, TGV, NLTV, NRL1, ASWMF, the proposed model shows the best performance in terms of PSNR and MSSIM.

## Conclusions

This paper introduces a novel variational regularization model to restore images contaminated by salt and pepper noise. Different from the very famous TVL1 model, the proposed model uses a nonconvex total variation $\hbox{TV}_q(0<q<1)$ as the regularizer, which enables the model to be more effective for edge-preserving. A first-order algorithm based on ADMM combining with MM scheme and proximity operator to solve this nonconvex minimization problem. In addition, a sufficient condition for the convergence of the proposed algorithm is provided. Numerical results demonstrate that the proposed model can effectively remove salt and pepper noise while preserving image edges and contours. Moreover, compared with TVL2, NTVL2, TVL1, TGV, NLTV, NRL1 and ASWMF models, the proposed model shows the best performance in terms of PSNR and MSSIM values. It yields about 0.5 dB PSNR and 0.06 MSSIM improvements against all compared models.

It should be point out that our nonconvex $\hbox{TV}_q$ regularization may lead to undesired artificial staircase in the restorations. In the future, we will focus on solving this problem by introducing some nonconvex high-order TV regularization. In addition, the ADMM algorithm used in this paper cannot guarantee to find the global optimum of the model. Therefor, another successive research is to combine some other algorithms, such as nature-inspired heuristic algorithms^51–54^, arithmetic optimization algorithms^55^.

## Data Availability

The data that support the findings of this study are available on request from the corresponding author.
